# The effects of repetitive transcranial magnetic stimulation in older adults with mild cognitive impairment: a protocol for a randomized, controlled three-arm trial

**DOI:** 10.1186/s12883-019-1552-7

**Published:** 2019-12-16

**Authors:** Joy L. Taylor, Benjamin C. Hambro, Nicole D. Strossman, Priyanka Bhatt, Beatriz Hernandez, J. Wesson Ashford, Jauhtai Joseph Cheng, Michael Iv, Maheen M. Adamson, Laura C. Lazzeroni, Margaret Windy McNerney

**Affiliations:** 1 US Department of Veterans Affairs (VA) Palo Alto Health Care System (151Y), Sierra-Pacific Mental Illness Research Education Clinical Center (MIRECC), 3801 Miranda Avenue, Palo Alto, CA 94304-1207 USA; 2 Department of Psychiatry and Behavioral Sciences, Stanford University, School of Medicine, Stanford, CA USA; 3 War Related Illness and Injury Study Center (WRIISC), VA Palo Alto Health Care System, Palo Alto, CA USA; 4 Department of Radiology, Division of Neuroimaging and Neurointervention, Stanford University Medical Center, Stanford, CA USA; 5 Defense and Veterans Brain Injury Center and Polytrauma (DVBIC), VA Palo Alto Health Care System, Palo Alto, CA USA

**Keywords:** Mild cognitive impairment, Transcranial magnetic stimulation, Prefrontal cortex, Parietal cortex, Neuroimaging, Aging, Alzheimer disease

## Abstract

**Background:**

Mild Cognitive Impairment (MCI) carries a high risk of progression to Alzheimer’s disease (AD) dementia. Previous clinical trials testing whether cholinesterase inhibitors can slow the rate of progression from MCI to AD dementia have yielded disappointing results. However, recent studies of the effects of repetitive transcranial magnetic stimulation (rTMS) in AD have demonstrated improvements in cognitive function. Because few rTMS trials have been conducted in MCI, we designed a trial to test the short-term efficacy of rTMS in MCI. Yet, in both MCI and AD, we know little about what site of stimulation would be ideal for improving cognitive function. Therefore, two cortical sites will be investigated in this trial: (1) the dorsolateral prefrontal cortex (DLPFC), which has been well studied for treatment of major depressive disorder; and (2) the lateral parietal cortex (LPC), a novel site with connectivity to AD-relevant limbic regions.

**Methods/design:**

In this single-site trial, we plan to enroll 99 participants with single or multi-domain amnestic MCI. We will randomize participants to one of three groups: (1) Active DLPFC rTMS; (2) Active LPC rTMS; and (3) Sham rTMS (evenly split between DLPFC and LPC locations). After completing 20 bilateral rTMS treatment sessions, participants will be followed for 6 months to test short-term efficacy and track durability of effects. The primary efficacy measure is the California Verbal Learning Test-II (CVLT-II), assessed 1 week after intervention. Secondary analyses will examine effects of rTMS on other cognitive measures, symptoms of depression, and brain function with respect to the site of stimulation. Finally, selected biomarkers will be analyzed to explore predictors of response and mechanisms of action.

**Discussion:**

The primary aim of this trial is to test the short-term efficacy of rTMS in MCI. Additionally, the project will provide information on the durability of cognitive effects and potentially distinct effects of stimulating DLPFC versus LPC regions. Future efforts would be directed toward better understanding therapeutic mechanisms and optimizing rTMS for treatment of MCI. Ultimately, if rTMS can be utilized to slow the rate of progression to AD dementia, this will be a significant advancement in the field.

**Trial registration:**

Clinical Trials NCT03331796. Registered 6 November 2017, https://clinicaltrials.gov/ct2/show/NCT03331796. All items from the World Health Organization Trial Registration Data Set are listed in Appendix A.

**Protocol version:**

This report is based on version 1, approved by the DSMB on 30 November, 2017 and amended on 14 August, 2018 and 19 September, 2019.

## Introduction

The goal of this study is to test the efficacy of repetitive Transcranial Magnetic Stimulation (rTMS) as a treatment for amnestic Mild Cognitive Impairment (MCI). MCI describes a clinical entity between healthy cognitive aging and dementia, in which individuals are cognitively impaired but do not meet the full criteria for dementia [1, 2]. Amnestic MCI (aMCI), in which individuals experience mild memory impairment with or without impairments in other cognitive functions [3–5], can presage dementia due to Alzheimer’s Disease (AD), though not every person with aMCI will progress to AD dementia [6]. Among adults aged 65 or older, 16 to 20% are likely to fit the overall entity of MCI [3, 7]. Once diagnosed, the annual rate of conversion to dementia averages 12% per year [7, 8]. MCI is an important public health concern due to its prevalence, risk of progression to dementia, and lack of effective treatment.

During the past decade, clinical trials testing whether cholinesterase inhibitors could slow the rate of conversion from MCI to dementia, or at least provide a temporary boost to cognitive performance, yielded disappointing results [9–11]. Reviews of cholinesterase inhibitors, as well as a meta-analysis involving well over 4000 MCI patients, concluded that the low efficacy of cholinesterase inhibitors is outweighed by their adverse effects (e.g. gastrointestinal discomfort, unusual dreams, and leg cramps) [12–15]. A more specific augmentation of the cholinergic system is currently being tested in a 24-month clinical trial of transdermal nicotine (NCT02720445). This clinical trial follows a 6-month pilot trial that demonstrated nicotine improved the primary efficacy measure, attention, in older adults with MCI (standardized mean difference; SMD = 0.78) [16]. To date, no pharmacological treatment has been approved for MCI, and no non-pharmacological treatment has shown satisfactory efficacy for MCI. Systematic reviews of treatments for MCI [12, 13, 17] indicate that the treatment investigated was ineffective; or if the treatment showed promise of efficacy, the finding still needs to be independently replicated; or the clinical trial was underpowered [12]. Presently, healthy behaviors—particularly exercise, control of cardiovascular risk factors, and cognitive/social activities—are the recommended approaches for coping with MCI. In summary, aside from general health recommendations, there are currently no effective treatments for MCI.

At the leading edge of innovative and safe pilot treatments for improving cognitive function in older adults is rTMS [18, 19], a noninvasive brain stimulation (NIBS) technique. In the Discussion, we outline the therapeutic rationale for rTMS with respect to targeting aMCI abnormalities in brain function. Here, we summarize emerging findings on the efficacy of multiple-session rTMS for improving cognitive function in MCI. Two sham-controlled, blinded trials involving nondemented older adults with MCI have been published [20, 21]. These two trials, which follow up on promising studies showing improvements in cognitive function in patients with mild to moderate Alzheimer’s disease (AD) dementia [22–27], employed roughly similar rTMS treatment protocols, in which 10 Hz high-frequency “excitatory” rTMS [28] was applied over the left dorsolateral prefrontal cortex (DLPFC) for 10 weekday sessions. The first rTMS-MCI trial, involving 34 MCI patients, used a parallel groups design [20]. Participants in the active group received 10 Hz rTMS delivered as 2000 pulses per session (5-s train duration; 25-s inter-train interval (ITI); 110%MT). Compared to sham, rTMS treatment significantly improved the primary outcome, scores on the Rivermead Everyday Memory Test. This improvement in memory performance was sustained at the 1 month follow-up time point (SMD = 0.78). A second rTMS trial, involving 9 patients with apathy and MCI used a cross-over design [21]. Participants received 10 Hz rTMS (4-s train duration, 26-s ITI; 120%MT; 3000 pulses per session) for 10 weekday sessions in the active condition. After a 4-week treatment-free period, the cohorts crossed over and repeated the 10 weekday sessions. Participants showed significant improvement in the primary outcome, the Apathy Evaluation Scale (*p* = .045), after active treatment compared to sham. Three of the six secondary cognitive outcomes also showed significant improvement; these were the Modified and original Mini-Mental State total scores and the Trail Making Test, Part A time score.

Supplementing the scant literature on the efficacy of rTMS for MCI are three additional studies on rTMS for memory improvement: [1] a controlled cross-over trial in “prodromal AD” [29] (i.e., the participants had mild memory loss and were positive for amyloid and tau AD biomarkers [30]) [2]; a single-session rTMS experiment in aMCI [31]; and [3] a single-session rTMS experiment involving older adults with below-normal memory performance [32]. In the single-session experiments, 5 Hz high-frequency rTMS applied over the left DLPFC for 5 min significantly improved face-name associative memory performance relative to the sham treatment [32], and 1 Hz low-frequency rTMS (i.e. “inhibitory” rTMS [33]) applied over the right DLPFC for 10 min following the study of non-verbal stimuli led to enhanced recognition accuracy [31]. In the multi-session sham-controlled trial in “prodromal AD” [29], participants (*n* = 14) received 20 Hz excitatory rTMS (2-s train duration; 28-s ITI; 100% resting MT; 1600 pulses per session) over the precuneus for 10 weekday sessions in the active rTMS condition. There was a 2-week washout period before crossing over to the other condition. The authors hypothesized that stimulation of the precuneus region of the medial parietal lobe would selectively improve episodic memory. Indeed, participants showed significant improvement in memory, as measured by the Rey Auditory Verbal Learning delayed recall score, following active rTMS; other cognitive scores were not significantly changed [29].

Together, these preliminary findings suggest that rTMS can enhance memory and cognition in MCI patients. Of nine other randomized, controlled rTMS studies in patients with AD dementia [22–27, 34–36], eight studies demonstrated significant improvement of cognitive function [22–27, 35, 36]. Yet, we know little about how long the effects of rTMS last, which is key to knowing how frequently rTMS would need to be repeated to slow or prevent progression of impairment. The limited follow-up data published so far come primarily from AD trials, and indicate that five or more sessions of rTMS can produce cognitive benefits lasting up to 4 months [23–25]. Moreover, it is not clear what site for non-invasive stimulation is ideal, given the pattern of neurodegeneration and cognitive deficits in MCI and AD [37]. In the rTMS studies to date on treatment of MCI and AD, the site most frequently targeted was the DLPFC, the same stimulation site used for treatment of depression. Is the DLPFC truly the ideal stimulation site for MCI and AD? Stimulation of the LPC gained proof of concept in a study of healthy young adults, in which 5 daily sessions of rTMS over the LPC led to significant improvement of associative memory [38]. Ultimately, stimulating both prefrontal and parietal regions might provide superior clinical outcomes given the profound effects of AD on widespread interconnected brain regions [39, 40]. Clearly, a greater understanding of how rTMS applied over a cortical site leads to changes in cognitive and brain function is important in deciding whether one site is ideal and sufficient, or whether stimulating multiple sites could be superior [41]. The current protocol is designed to rigorously test the short-term efficacy of rTMS in aMCI, while also commencing to address crucial questions about choice of stimulation site and durability of effects.

### Study aims

The primary aim is to test the hypothesis that patients receiving active rTMS will show more improvement in memory at the 1-week post-intervention assessment than the sham-control group. Secondarily, we aim to:

1. Assess the durability of rTMS effects on memory over a 6-month follow-up period, which is longer than assessed in previous studies;
2. Examine effects of rTMS on behavior and brain function related to the site of stimulation, which is a novel approach in the AD spectrum;
3. Explore patient characteristics that could be useful in identifying who responds preferentially to rTMS or to a particular stimulation site.

## Methods/design

### Study design and overview

**Fig. 1:**
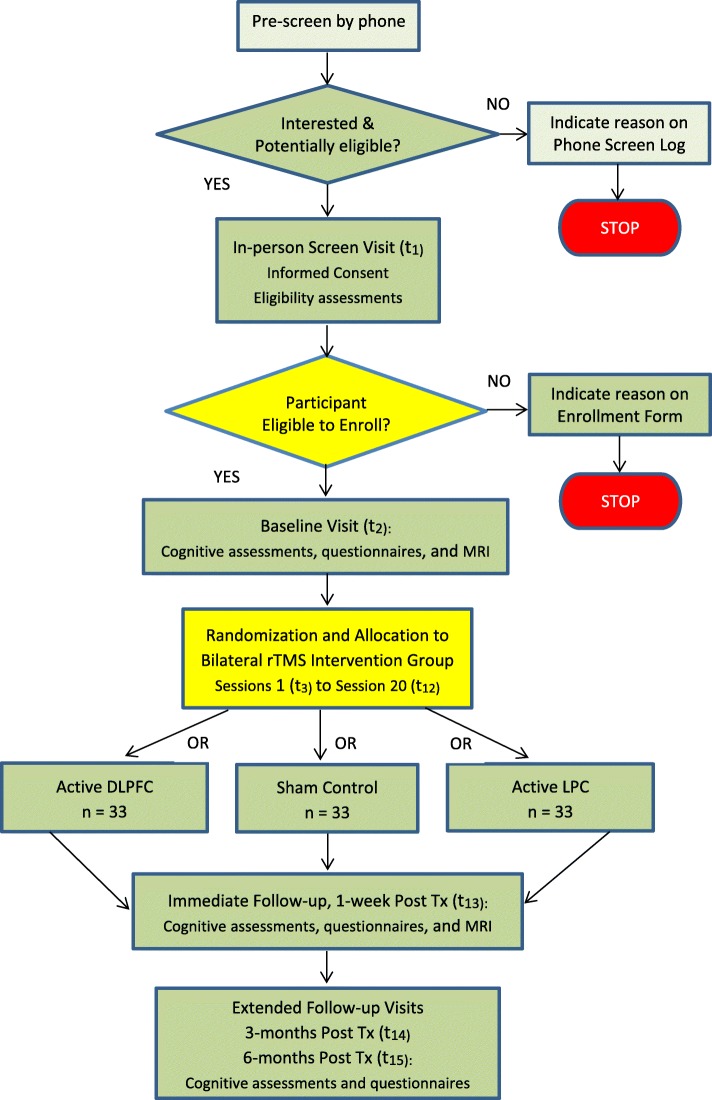
A summary of the study design and participant flow through the study

Our hypotheses regarding the effects of rTMS on memory in persons with aMCI will be tested in a randomized, 2-stimulation-site, sham-controlled, 3-arm double-blind design, in which bilateral rTMS is delivered as: [1] active rTMS of the DLPFC, [2] active rTMS of the Lateral Parietal cortex (LPC), or [3] sham/inactive rTMS (evenly split between each coil location). Participants will receive 20 treatment sessions. Behavioral measures will be collected at baseline, at 1-week post-intervention, and at two subsequent follow-up visits at 3 and 6 months. In addition to the primary efficacy measure of memory, secondary measures of global cognitive function, language, executive control, depressive symptoms, and everyday function will be obtained. Resting-state functional Magnetic Resonance Imaging (rs-fMRI) scans will be acquired at baseline and 1-week post-intervention to examine effects of rTMS on functional connectivity. The rTMS-MCI project is designed to ultimately include data from 99 aMCI participants (*n* = 33 per group), with measures of cognitive efficacy, brain function, and predictors of response to rTMS. The study site is the Stanford/VA Aging Clinical Research Center, an outpatient clinical research center located at the VA Palo Alto Health Care System near the Stanford University School of Medicine. The trial design and study flow are shown graphically in Fig. 1.

### Participants

The target population is individuals with single- or multi-domain amnestic MCI (aMCI). For this study, we use the Alzheimer’s Disease Neuroimaging Initiative (ADNI-3) characterization of aMCI, which uses a combination of clinical and neuropsychological assessments to classify participants into categories of normal cognition, MCI, or AD dementia [42].

### Enrollment

#### Inclusion criteria

Participants are eligible for randomization if they fulfill the following inclusion criteria:

1. Age 55–90 years inclusive;
2. Diagnosed with aMCI as defined by ADNI-3 [42];
  - Participant must express a subjective memory concern as reported by participant or recalled by study partner or clinician.
  - Below normal memory function documented by scoring below the education-adjusted cutoffs on the Logical Memory II subscale (Delayed Paragraph Recall, Story A) from the Wechsler Memory Scale–Revised (the maximum score is 25): a) less than 11 for 16 or more years of education; b) less than or equal to 9 for 8–15 years of education; c) less than or equal to 6 for 0–7 years of education.
  - Mini-Mental State Exam score between 24 and 30 (inclusive). (Exceptions may be made for subjects with less than 8 years of education at the discretion of the project director).
  - Clinical Dementia Rating = 0.5. Memory Box score must be at least 0.5.
  - General cognition and functional performance sufficiently preserved such that a diagnosis of dementia cannot be made by the physician at the time of the screening visit.
3. Stable medications (including any dementia-related meds) for at least 4 weeks prior to the Baseline visit;
4. Geriatric Depression Scale score less than 6;
5. Ability to obtain a motor threshold, determined during the screening process;
6. Study partner available; living situation enables attendance at clinic visits;
7. Visual and auditory acuity adequate for neuropsychological testing;
8. Good general health with no diseases expected to interfere with the study, as determined by the referring Memory Clinic Physician or the rTMS Study Physician;
9. Participant is not pregnant or of childbearing potential;
10. Modified Hachinski Ischemic score less than or equal to 4;
11. Agree to DNA extraction for single nucleotide polymorphism (SNP) genotyping;
12. Able to understand study procedures and comply with them for the entire length of the study.

#### Exclusion criteria

1. Prior exposure to rTMS within the past 12 months;
2. Magnetic field safety concern such as a cardiac pacemaker, cochlear implant, implanted device in the brain, or metal fragments or foreign objects in the eyes, skin or body;
3. Any significant neurological disease other than suspected incipient Alzheimer’s disease;
4. Unstable cardiac disease or recent (< 3 months previous) myocardial infarction. Any significant systemic illness or unstable medical condition that could lead to difficulty with protocol adherence;
5. History of epilepsy or repetitive seizures, as determined by patient report or chart review;
6. History of a medical condition or current use/abuse of medications and substances that increase the risk of a seizure, specifically:
  - Traumatic brain injury within the past 2 months;
  - Unable to safely withdraw, at least 4 weeks prior to Baseline, from medications that substantially increase the risk of having seizures (for example, theophylline, clozapine, and methylphenidate; the complete list of Exclusionary Medications is available from the corresponding author).
  - Current or past history of a mass lesion, cerebral infarct, or other non-cognitive active neurological disease that would increase the risk for seizure.
  - Stimulant abuse within the previous 90 days. Cocaine and abuse of amphetamine and methylphenidate are associated with an increased risk of seizures;
  - Major depression or bipolar disorder (DSM-IV) within the past 1 year, or psychotic features within the last 3 months that could lead to difficulty with protocol adherence;
7. Taking sedative hypnotics or medications with anti-cholinergic properties and unable to withdraw at least 4 weeks prior to Baseline;
8. Current alcohol or substance abuse (not including caffeine or nicotine) within the past 1 year, as determined by chart review, participant or study partner report, or greater than “moderate” alcohol use defined by the Quantity-Frequency-Variability Index [43];
9. Any contraindications for MRI studies (e.g. severe claustrophobia, pregnancy, pacemaker, weight above 160 Kg maximum allowed by MRI scanner);
10. Participation in another concurrent clinical trial;
11. Inability or unwillingness of individual or legal representative to give written informed consent.

#### Recruitment strategies

Multiple strategies will be used to achieve adequate enrollment and reach the target sample size, including the following: (1) targeted recruitment via memory clinics that are affiliated with the Stanford University School of Medicine; (2) collaboration with relevant online research registries such as the Alzheimer’s Prevention Registry and the Brain Health Registry; and (3) flyer postings at the VA Palo Alto Health Care System’s facilities; (4) networking with local neurologists, psychiatrists and neuropsychologists; and (5) direct-mail advertising to individuals based on age and geography.

#### Determination of study eligibility

As illustrated in in Fig. 1, determination of study eligibility is a two-stage process. First, interested individuals are pre-screened by telephone using an IRB-approved screening script. Potentially eligible participants are then invited to come to the clinic for a Screening Visit (t_1_) that begins with obtaining written informed consent. Next, eligibility assessments are performed, as described in the “Measures” section. If the participant meets all eligibility criteria, behavioral measures and an MRI are acquired at a separate Baseline Visit (t_2_). Following completion of the Baseline procedures, the Database Manager randomizes the participant to one of the three intervention arms. More details on randomization and double-blinding are described under “Randomization.” Briefly, a unique 6-digit number is assigned to the participant; when this 6-digit number is entered into the interface of the TMS device, the software enables the device and an unmarked active-placebo (A/P) coil to deliver the appropriate intervention (active or sham) to that participant.

### Intervention

#### rTMS stimulation protocol, parameters, and coil placement

The intervention is administered as 20 weekday sessions during a period of 2 to 4 weeks. Typically, a participant will have a morning session and an afternoon session. The intervention uses rTMS stimulation procedures that have proven evidence of efficacy to improve cognitive function in AD dementia, based on two meta-analyses [44, 45]. Specifically, high-frequency (10 Hz) rTMS is delivered bilaterally, as this procedure was significantly effective in improving the cognition of AD patients [44]. Stimulation will be applied over the right hemisphere first, and immediately followed by the left hemisphere, following the procedure of two previous bilateral stimulation trials [23, 24]. Coil placement is guided by a Localite neuronavigation system (Localite GmbH, Bonn, Germany) using the MRI data acquired at the baseline visit. By using neuronavigation, we aim to optimize subjects’ clinical responses, irrespective of whether the subject is assigned to DLPFC or LPC stimulation. DLPFC participants will be stimulated on the left at the MNI coordinate x = − 38, y = 44, z = 26, which was identified by Fox et al. [46] as a DLPFC site associated with more efficacious outcomes in rTMS treatment of depression. LPC participants will be stimulated on the left at the MNI coordinate x = − 47, y = − 68, z = 36, which was the MNI centroid used by Wang et al. [38] for fMRI-guided neuronavigation of rTMS; Wang et al. [38] successfully modulated cortical-hippocampal connectivity and improved associative memory performance in healthy, young adults. For both DLPFC and LPC participants, stimulation of the right hemisphere is targeted to the corresponding coordinate on the right (e.g. x = 47, y = − 68, z = 36 to target the right LPC).

The same stimulation parameters are used to deliver both of the active interventions (DLPFC and LPC); specifically, a pulse frequency of 10 Hz with a 4-s train duration (40 pulses) and 11-s ITI. During each session, 2000 pulses will be applied over each hemisphere at 120% of the participant’s resting motor threshold, which is individually measured for each hemisphere. In summary, this 10 Hz stimulation protocol delivers 4000 pulses per session and up to 8000 pulses per day, with a total of 80,000 pulses over 2- to 4-week period.

#### Equipment and masking procedures

The intervention is delivered using a MagPro X100 magnetic stimulator. The Cool-B65-A/P coil system creates auditory “clicks” for sham intervention that match the click sounds of active rTMS. Additionally, for each session, whether sham or active, each participant wears scalp electrodes through which a low-voltage, low electric current (2–6 mA at no more than 100 V) is passed in order to provide cutaneous stimulation that mimics the sensation of actual rTMS. Additional details are provided in Mi et al. [47] and the TMS operator manual developed for this protocol. Note: Additional file 1: “List of supplementary documents” provides a list of rTMS manuals that can be obtained from the authors.

#### Intervention discontinuation

The study intervention will be discontinued for a participant if:

1. The participant has a seizure;
2. There are signs or reports of inability to tolerate the intervention, or if the study physician feels it is in the participant’s best interest to discontinue with the intervention;
3. Or, there is any other health or safety concern that contraindicates continuation based on the judgment of the study physician.

Such participants will be encouraged to return for post-intervention follow-ups. Except for the MRI scan, all other outcome measures, adverse effect (AE) information and safety assessments would be collected during the follow-up period.

### Measures

Additional file 2 Table S1 lists the schedule of all study assessments, including eligibility, safety, and outcome measures. The assessments are described briefly below:

### Eligibility assessments

#### Assessment of aMCI

During the screening visit, the study coordinator assesses memory and global cognitive function using the Logical Memory (Story A) portion of the Wechsler Memory Scale-Revised [48] and the Mini-mental Status Exam (MMSE) [49]. Independently of the study coordinator, a certified rater assesses the participant’s functional competence using the Clinical Dementia Rating scale (CDR) [50]. To screen for depression and ensure that a mood disorder is not a primary cause of memory impairment, the participant completes the Geriatric Depression Scale (GDS) [51]. To confirm the presence of subjective memory decline, the participants fills out the Cognitive Change Index questionnaire [52]. A physician interviews the participant and provides a modified Hachinski rating [53] to ensure that cerebrovascular disease is not a primary cause of memory impairment. The classification criteria for aMCI that are used in this clinical trial are listed above under “Inclusion criteria.”

### Safety, blinding, and TMS acceptability assessments

#### TMS/MRI safety assessments

During the screening phase and prior to rTMS or MRI sessions, participants answer questionnaires regarding their safety in undergoing rTMS intervention or an MRI (surgical history, epilepsy history, presence of metal or cardiac pacemaker, etc.). Participants also undergo a detailed history, physical, and neurological screening exam by a licensed physician whose approval must be given before enrollment. Vital signs as well as medication, sleep, and substance use history are measured throughout the study to ensure participant safety.

Collection and monitoring of any AEs begins at the time the participant signs the informed consent form and continues throughout the 6-month post-intervention follow-up phase. Specific procedures for collecting, assessing, reporting, and managing solicited and spontaneously reported are described in the detailed study protocol, which is available from the authors. (See Additional file 1: “List of supplementary documents.”) The PI and research team will notify the Data and Safety Monitoring Board (DSMB) and the NIA Program Official of all Serious Adverse Events within 24 h of study staff’s knowledge of the event. All AEs will be reported annually or more often to the DSMB, Stanford IRB, and NIA staff. Oversight by the DSMB is described further in the “Monitoring” section of the Methods.

### Outcome measures

#### TMS blinding and acceptability questionnaires

Before receiving and after completing the intervention, both the participant and the TMS operator are asked to guess the participant’s group assignment. At the end of intervention, the participant answers open-ended questions about treatment satisfaction and the tolerability of rTMS.

### Outcome measures

#### Primary outcome measure

The primary outcome is change in memory performance, observed 1 week following intervention (t_13_). Memory is assessed using the California Verbal Learning Test (CVLT-II) [54]; the primary dependent variable is the CVLT-II Total score summed over Trials 1–5 (possible range 0–80; higher values represent a better outcome). The CVLT is a two-list memory task that assesses multiple aspects of verbal learning and episodic memory. List A of the CVLT-II contains 16 concrete words; four words each from four categories (animals, vegetables, ways of traveling, and furniture). List A is presented for 5 learning trials. After presentation and recall of another List (B), a short-delay free recall and cued recall of list A is performed. After a 20-min delay filled with nonverbal testing, long-delay free recall and cued recall are assessed, followed by a yes/no recognition test for List A.

Use of the CVLT-II Total (T1–5) score as the primary outcome should allow for a wide range of scores that are neither at floor nor at ceiling level, based on previous research [55–57]. The CVLT-II Total 1–5 score also has high diagnostic validity. The CVLT-II Total 1–5 score had the highest predictive value for MCI-to-dementia conversion [58] and was the best variable for distinguishing MCI from normal cognitive function [59], in comparison to other measures of episodic memory. Additional advantages of the CVLT-II include: excellent retest reliability (e.g. Total 1–5 reliability: r = .82); the availability of an alternate test form (Total 1–5 alternate-form reliability: r = .79), and relevant normative data. In summary, we selected the CVLT-II for reasons related to sensitivity, validity, reliability, and, as discussed in the next section, its potential utility for detecting treatment-response differences related to the cortical site of rTMS stimulation.

#### Secondary behavioral outcome measures

A variety of secondary measures are being collected to compare and contrast the effects of DLPFC and LPC stimulation. First of all, the clustering and recall scores of the CVLT-II will provide key measures of memory encoding and retrieval, which in turn relate to the distinct roles that DLPFC and LPC regions have in supporting these processes [60]. The other secondary outcome measures were selected on the basis of previous studies of rTMS in MCI and AD that reported statistically significant improvements in global cognitive function [22–24, 61, 62], language [25–27], instrumental activities of daily living [23], and a reduction of depressive symptoms [23]. The following secondary behavioral measures are collected at 4 timepoints: baseline (t_2_), 1-week post intervention (t_13_), 3-months post intervention (t_14_); and at 6-months post intervention (t_15_):

#### Cognitive measures

1. Secondary CVLT-II [54] verbal episodic memory measures of interest are: semantic clustering (chance-adjusted) Trials 1–5; and the short- and long-delay free recall scores (0–16; higher values represent a better outcome). Our working hypothesis is that DLPFC stimulation could result in a higher level of semantic clustering compared to LPC stimulation. By contrast, LPC stimulation could result in improved recall, assessed by the CVLT-II short- and long-delayed recall scores.
2. Global cognitive function is measured using the Montreal Cognitive Assessment (MoCA) [63] Total score (0 to 30; higher values represent a better outcome).
3. Visuospatial episodic memory is measured using the Brief Visuospatial Memory Test–Revised (BVMT-R) [64], Trials 1–3 Total raw score (0–36; higher values represent a better outcome).
4. Language abilities are measured using: Category Fluency [65], total number of correct responses in 60 s (higher values represent a better outcome); and 42-item Boston Naming Test (BNT) [66], Total score (0–42; higher values represent a better outcome).
5. Visuoconstructional function is measured using the Rey-Osterrieth Complex Fig [67, 68]., Copy score (0–36; higher values represent a better outcome).
6. Speed of processing and executive control are measured by the Trail Making Test, Parts A and B (number of seconds to complete; lower values represent a better outcome) [69, 70].

### Functional questionnaires

The participant and study partner will be asked to independently fill out the Everyday Cognition Scale [71], which asks about perceived changes in performance of everyday tasks (ECog Total score, range: 39–156; higher values represent a worse outcome). The study partner will also complete the Functional Activities Questionnaire (FAQ) [72], which assesses the participant’s level of independence in performing everyday activities (FAQ Total score, range: 0–30; higher values represent a worse outcome).

### Depressive symptoms

The participant will complete the 15-item GDS [51]. (Total score, range: 0–15; higher values represent a worse outcome). Because the GDS is also an eligibility assessment, it is obtained prior to baseline at t_1_; the t_1_ value will be treated as the baseline timepoint.

#### Secondary neuroimaging outcomes

MRI scans are acquired at baseline (t_2_) and 1-week post intervention (t_13_) on a 3 Tesla scanner (GE Healthcare, Chicago, Illinois). During each 40 min scan, the following five sequences are acquired: anatomical 3D T1-weighted gradient echo sequence [sagittal T1-weighted inversion-recovery-prepared, fast spoiled gradient recalled BRAin VOlume (BRAVO) imaging, 1 mm^3^ voxel size], rs-fMRI [3 mm^3^ voxel size, 9 min scan duration, using simultaneous multi-slice (SMS) acquisition]; 3D T2-weighted FLAIR (fluid-attenuated inversion recovery) sequence (sagittal imaging combined with an isotropic 3D fast spin echo acquisition, “CUBE”); axial non-contrast Arterial Spin Labeling (ASL) perfusion imaging; and diffusion-weighted echo-planar imaging (DW-EPI and epi2alt acquisitions, axial 2D, 25 directions for each acquisition). Functional connectivity metrics will be derived from the rs-fMRI scans for use in secondary and exploratory analyses.

#### Secondary biomarker variables

Plasma levels of brain-derived neurotropic factor (BDNF) are measured from fasting blood samples that are collected at the first t_3_ and last t_12_ morning intervention sessions to gain information about mechanisms of action of rTMS with respect to brain plasticity. Genomic DNA is acquired from blood at t_3_ to explore genetic predictors of response to rTMS. Additional file 4 describes procedures for the collection, processing, and storage of biological specimens.

### Randomization

Participants are randomized in a 2:1 ratio to active rTMS or sham at baseline. Within the active and sham groups, participants are randomized in a 1:1 ratio to the two unmasked cortical sites. The method of random assignment to treatment (active vs. sham) and to cortical site (DLPFC vs LPC) is determined by a computer-generated random sequence. An adaptive randomization scheme is being used so that equal numbers of participants are randomized to each of the 3 treatment groups within blocks of every 6 participants. For each block, 4 active and 2 sham treatment numbers are randomly assigned. To ensure proper allocation of participants, a block of 6 participants consists of: 2 active DLPFC, 2 active LPC, 1 sham DLPFC and 1 sham LPC.

### Blinding / masking

To accomplish double-blinding, the study’s database manager randomizes each participant to treatment and maintains the list of randomization codes in an encrypted restricted-access file; only the Database Manager knows the encryption key. Thus, study staff—including the principal investigator, study coordinator, and TMS operator—and participants are blinded to group assignment.

### Statistical analysis plan and sample size calculations

Outcome measures will be analyzed at study completion by the database manager under the supervision of a Ph.D. biostatistician. For the purposes of analyses, there will be 3 equally sized treatment groups of interest: 1) active DLPFC; 2) active LPC; and 3) sham. The sham group is comprised of all subjects assigned to the sham group, regardless of the position of the sham coil.

#### Primary analysis

The primary hypothesis is that participants receiving active rTMS will show more improvement in memory than the sham group at the 1-week post-intervention assessment. An Intention-to-treat (ITT), Analysis of Covariance (ANCOVA) will be run on the CVLT-II Trials 1–5 Total raw t_13_ score using the baseline CVLT-II score as a covariate. ITT includes all randomized patients who started at least 1 intervention session. Testing the primary hypothesis will involve two statistical tests: Test 1 will compare active DLPFC to sham; Test 2 will compare active LPC to sham. The two tests will be conducted at a 2-sided alpha level of 0.05, adjusted for two tests.

##### Statistical power

We powered the study to detect a moderately large effect size (Cohen’s d = 0.80) of either rTMS treatment on the primary outcome. Moderately large effect sizes of 1.0, 0.80, and 0.80 were reported in two meta-analyses of rTMS studies in AD [44] [45] and one rTMS trial in MCI (which used unilateral rTMS [20]. With a total sample size of 99 participants, our study is estimated to have 80% power with a multiple testing penalty for two tests. For follow-up analyses of differences related to the site of stimulation, this sample size will provide 80% power to detect a large difference (d = 1.00). For all analyses, we will convey practical significance by reporting effect sizes and confidence intervals, in addition to reporting statistical significance.

#### Secondary analyses

For all analyses, parameter estimates will be reported for Model 1: active DLPFC vs. sham, and for Model 2: active LPC vs. sham.

##### Durability

The durability of rTMS effects on memory over the entire study period will be examined using mixed-effects growth curve modeling [73]. The magnitude of treatment effects at the 3-mos and 6-mos time points will be reported. Our working hypothesis is that an effect of rTMS on the primary outcome, the CVLT-II Trials 1–5 Total score, will be sustained up to 3 months.

##### Behavioral differences related to the site of brain stimulation

The effect of rTMS effects on the secondary CVLT-II memory scores and other cognitive outcomes, on depressive symptoms, and on functioning will be examined using mixed-effects growth curve modeling [73]. Separate models will be fit for each secondary outcome. To aid in contrasting the magnitude of effects of DLPFC vs. LPC stimulation, parameter estimates and 95% confidence intervals will be reported for each stimulation site. Potential differences in the magnitude of effects on semantic clustering and delayed recall sub-scores of the CVLT-II will be particular interest.

##### Functional connectivity

With respect to therapeutic effects of rTMS on functional connectivity, changes in connectivity *within* the posterior Default-mode network (DMN), and changes *between* the DMN and networks that support task engagement [74, 75] are of particular interest. This is because of reports that aMCI patients, compared to cognitively unimpaired older adults, show 1) abnormal connectivity within the DMN, as well as 2) abnormal interactions between the DMN and other networks, in particular the Salience network and the Central Executive network (CEN) [76]. To examine effects of rTMS on functional connectivity, two sets of analyses will be performed on the baseline (t_2_) and 1-week post intervention (t_13_) rs-fMRI data: [1] rTMS effects on connectivity within the DMN. The aim of these analyses is to discover the extent to which rTMS restores connectivity of the posterior cingulate cortex (PCC) within regions of the DMN; and [2] rTMS effects on between-network connectivity. We will examine if rTMS reduces abnormally strong functional connectivity between the CEN and DMN, i.e. makes the two networks more anti-correlated. Change in connectivity from baseline to 1-week post intervention will be computed, and contrasts will be made between each active rTMS group and the sham group, and between the LPC and DLPFC groups. Appropriate corrections for multiple comparisons will be made to provide an overall alpha probability of a type I error of < 0.05.

#### Exploratory analyses

Change in BDNF levels from the beginning to the end of intervention will be examined using ANCOVA. Our working hypothesis is that BDNF levels will increase more in either active rTMS group, compared to that in the sham group. Though a direct link between rTMS and synaptic plasticity remains to be demonstrated in humans, an effect of rTMS on BDNF levels would be consistent with a beneficial effect on brain plasticity. Finally, baseline functional connectivity, genetic, and selected clinico-demographic variables will be explored as potential moderators of response to rTMS. The results of these exploratory analyses are useful toward generating hypotheses directed toward ultimately identifying who responds preferentially to rTMS or to a particular stimulation site.

### Dissemination of research results

Following completion of the study, a manuscript will be prepared for the primary outcome (change in CVLT-II performance observed 1 week following intervention) and for the secondary memory-related outcomes, such as the durability of effects of rTMS on memory over the study period. Additional manuscripts may be prepared to report on the effects of rTMS on other secondary behavioral outcomes (cognitive function and mood), and secondary neuroimaging and biomarker variables. Every manuscript will be reviewed and approved by the protocol director, key personnel, database manager, and all other co-authors prior to submission. Each participant will receive a summary of the study results along with revelation of their respective treatment group.

### Monitoring

#### Data safety monitoring board (DSMB)

An independent, external DSMB has been assembled to monitor participant safety, the progress of the study, and the quality of data collection. Each DSMB member—3 psychiatrists and 1 biostatistician—is an expert in the area of TMS, clinical trials, and/or MCI. The DSMB meets twice annually, typically by teleconference call. The DSMB determines when they should be un-blinded to treatment assignment for the reviewing of AEs. The DSMB advises the PI and funder whether the study should continue or be stopped. The DMSB or funder will determine if a planned interim analysis should be conducted. All protocol modifications are first approved by the DSMB, and then submitted to the IRB for its approval. An modification that affects participant activities or risk-to-benefit ratio will be incorporated into the informed consent form documents and communicated to participants. The DSMB will discharge itself from its duties when the last participant completes the study.

## Discussion

There is increasing evidence from clinical trials that rTMS can improve cognitive function in older adults with MCI [20, 21, 31], prodromal AD [29] and probable AD dementia [22–27, 35, 36]. These trials involved relatively small sample sizes and limited follow-up after intervention. Some studies did not have the benefit of a convincing sham coil system. The current protocol is designed to achieve rigorous, reproducible methods to test the efficacy of rTMS to improve memory in aMCI, while commencing to address essential questions of the duration of therapeutic effects and selection of stimulation site.

To our knowledge, this would be the first study to investigate differences between the effects of rTMS applied over a DLPFC versus an LPC site within the same study design. Stimulating the DLPFC could have effects that would not be achieved with LPC stimulation, such that stimulating the DLPFC may differentially improve executive function, encoding of new information, and depressive symptoms. Conversely, stimulating the LPC may differentially improve memory retrieval and retention—the core cognitive domain of aMCI.

This would be the first clinical trial of rTMS in aMCI to measure changes in brain function, as assessed with pre-post rs-fMRI and functional connectivity metrics. It is believed that rTMS offers a means to modulate the brain’s large-scale networks [77, 78] such as the DMN [79–81] and fronto-parietal /CEN [82] networks by positioning the rTMS coil over a cortical node of that network [83]. The effects of targeted stimulation are measurable weeks later in distal regions that are directly or indirectly connected with the cortical site stimulated [84]. Thus, precisely guided rTMS has the potential to improve brain network dynamics that are selectively abnormal in an individual patient or certain condition [85].

For patients with aMCI, abnormal connectivity involving the DMN is a therapeutic target for rTMS. Network nodes that are accessible include parietal cortical sites, which in turn show connectivity to other DMN regions in the limbic, medial temporal, and inferior temporal cortices. In aMCI, the PCC of the medial limbic lobe most consistently shows reduced resting-state activity [86]. The PCC is a key node within the DMN [87–89] and a cortical hub that dynamically participates in interactions of the DMN with other brain networks [88, 90]. An rTMS-fMRI study in healthy young adults showed that multiple sessions of rTMS, when applied to a site over the LPC, increased connectivity between the LPC and PCC, the LPC and hippocampus, and other regions within the posterior DMN [38]. Thus, stimulating the LPC of aMCI patients has the potential to help restore loss of functional connectivity within the posterior DMN and help restore the PCC’s connectivity with other DMN regions in the inferior temporal and medial temporal cortices (in particular, parahippocampal and fusiform gyri and the hippocampi).

On the other hand, rTMS delivered to the right or left DLPFC region can potentially improve network interactions between the CEN and DMN regions, based on rTMS studies involving adults with major depressive disorder [91] and healthy young adults [83]. Importantly, rs-fMRI studies of aging and the AD spectrum have revealed altered network interactions between the DMN and networks such as the CEN, which in turn correlate with lower cognitive performance [40, 92–94]. Thus, stimulating the DLPFC using rTMS has potential to improve DMN:CEN interactions in older adults with MCI. In summary, the present clinical trial aims to gain novel insights as to how rTMS could improve brain network function in aMCI patients by investigating changes in functional connectivity after noninvasive cortical stimulation.

If this clinical trial obtains a signal of improved memory performance or favorable changes in functional connectivity, the next step in the clinical development of rTMS for aMCI would be a multi-site Phase III trial with the inclusion of AD biomarkers. For individuals who are in the Alzheimer’s spectrum [95], the progression of AD from MCI to dementia reflects a cascade of neurodegenerative processes, of which amyloid-β facilitated tauopathy is a leading account of AD progression [96–98]. If it could be shown that neuromodulation via noninvasive brain stimulation could actually move an AD biomarker of pathologic Aβ, tau, or neuronal status in a positive direction, this would be a major step forward in developing disease-modifying treatments for AD.

The prediction of responsivity to noninvasive brain stimulation is a parallel goal of future clinical development. There is evidence that individuals with mild dementia show a better cognitive response to rTMS than do those with more severe dementia [23, 36]. The pattern of differential response to rTMS in relation to dementia severity is plausibly due to decline of inherent neuroplasticity, greater skull-to-cortex distance, or other factors. Conceivably, indices of baseline functional connectivity, genetic variants (e.g. BDNF and Apolipoprotein E (APOE), or more easily measured, low-cost clinico-demographic variables will ultimately help predict response to rTMS and other modes of noninvasive brain stimulation. There are currently no effective treatments for MCI, aside from general health recommendations. If rTMS can be effectively utilized in older adults with MCI to delay progression to dementia, this would represent a significant advance in the field of AD and related disorders.

## Study status

At the time of submission, the study has enrolled 24 study participants and has not completed participant recruitment or data collection.

### Supplementary information

**Additional file 1.** List of supplementary documents. The detailed study protocol (which includes details of data collection, AE monitoring, statistical considerations, and data management), exclusionary medications, and two rTMS manuals are available from the authors.

**Additional file 2: Table S1.** Schedule of enrollment, interventions, and assessments.

**Additional file 3.** Informed consent documents. Research consent forms for the clinical trial and for an ancillary study involving urine specimens.

**Additional file 4.** Biological specimens manual. Appendix of procedures for the collection, processing, and storage of biological specimens for the current trial.

## Data Availability

Not applicable. Data sharing is not relevant to this article. The study was still enrolling study participants at the time of submission; no datasets have been generated or analysed yet .

## Appendix

**Table 1:** World Health Organization (WHO) trial registration data set

Data Category	Information
Primary registry and trial identifying number	ClinicalTrials.gov NCT03331796
Date of registration in primary registry	6 November, 2017
Source of monetary support	The National Institute on Aging
Primary Sponsor	The National Institute on Aging
Contact for public queries	Joshua Teso (Study Coordinator) 650–852-3457 Joshua.Teso@va.gov
Contact for scientific queries	Joy Taylor, PhD joyt@stanford.edu Stanford/VA Aging Clinical Research Center 3801 Miranda Avenue (151Y) VA Palo Alto Health Care System Palo Alto, CA, USA 94304–1207
Public Title	Noninvasive Brain Stimulation for Mild Cognitive Impairment
Scientific title	Noninvasive Cortical Stimulation to Improve Memory in Mild Cognitive Impairment
Countries of recruitment	USA
Health condition or problem studied	Mild Cognitive Impairment (MCI), Other terms: Mild Neurocognitive Disorder, Memory Decline, Memory Loss, Memory Impairment
Intervention (s)	Repetitive transcranial magnetic stimulation (rTMS), 10 Hz, 20 sessions Active comparators (Arms 1 and 2): rTMS Bilateral Dorsolateral Prefrontal Cortex (DLPFC); rTMS Bilateral Lateral Parietal Cortex (LPC) Placebo comparator (Arm 3): Inactive sham coil treatment
Key Inclusion and Exclusion criteria	Inclusion: Age 55–90, amnestic MCI, stable medications, Geriatric Depression Scale score less than 6; Ability to obtain a motor threshold; Study partner available; Visual and auditory acuity adequate for neuropsychological testing; Good general health with no diseases expected to interfere with the study. Exclusion: Magnetic field safety concern such as a cardiac pacemaker, cochlear implant, metal fragments in the eyes, skin or body, or pregnancy; Any significant neurological disease other than suspected incipient Alzheimer’s disease; Unstable cardiac disease or recent myocardial infarction; Any significant systemic illness or unstable medical condition that could lead to difficulty with protocol adherence; History of epilepsy or repetitive seizure; History of a medical condition or current use/abuse of medications and substances that increase the risk of a seizure (e.g. recent traumatic brain injury, current use of stimulants), Known history of major depression or bipolar disorder within the past year; Current alcohol or substance abuse (not including caffeine or nicotine) within the past year.
Study type	Interventional Device / Phase II Allocation: randomized Intervention model: parallel assignment (3 arms) Masking: double blind Primary Purpose: treatment
Date of first enrollment	16 May 2018
Target sample size	99
Recruitment status	Recruiting
Primary outcomes	Change in memory, as measured by the California Verbal Learning Test-II (CVLT-II) Trials 1–5 Total raw score [Time Frame: Baseline, 1 week after completing the 20-session intervention]
Key Secondary outcomes	Behavioral outcomes include: Subscores of the CVLT-II, GDS, Everyday Cognition (ECog) Questionnaire, Montreal Cognitive Assessment (MoCA), tests of language, executive, and visuospatial function. Neuroimaging outcomes include: Change in functional connectivity metrics including comparison of DLPFC to LPC. Other biomarker outcomes: Change in brain-derived neurotropic factor (BDNF) plasma levels

## Abbreviations

A/P: Active-placebo
AD: Alzheimer’s Disease
ADNI: Alzheimer’s Disease Neuroimaging Initiative
AE: Adverse Event
aMCI: Amnestic Mild Cognitive Impairment
ANCOVA: Analysis of Covariance
APOE: Apolipoprotein E
ASL: Arterial Spin Labeling
BDNF: Brain-derived Neurotropic Factor
BNT: Boston Naming Test
BVMT-R: Brief Visuospatial Memory Test-Revised
CDR: Clinical Dementia Rating
CEN: Central Executive Network
CVLT-II: California Verbal Learning Test, second edition
DLPFC: Dorsolateral Prefrontal Cortex
DMN: Default Mode Network
DSMB: Data Safety Monitoring Board
DSM-IV: Fourth Edition of Diagnostic and Statistical Manual of Mental Disorders
ECog: Everyday Cognition Scale
FAQ: Functional Assessment Questionnaire
FLAIR: Fluid-Attenuated Inversion Recovery
fMRI: Functional Magnetic Resonance Imaging
GDS: Geriatric Depression Scale
ICNs: Intrinsically Connected Networks
IRB: Institutional Review Board
ITI: Inter-Train Interval
ITT: Intention-To-Treat
LPC: Lateral Parietal Cortex
MCI: Mild Cognitive Impairment
MMSE: Mini-Mental State Examination
MNI: Montreal Neurological Institute
MoCA: Montreal Cognitive Assessment
MRI: Magnetic Resonance Imaging
NIBS: Noninvasive Brain Stimulation
NIH: National Institutes of Health
PCC: Posterior Cingulate Cortex
PE/NE: Physical/Neurological Examination
rs-fMRI: Resting state functional Magnetic Resonance Imaging
rTMS: Repetitive Transcranial Magnetic Stimulation
SMS: Simultaneous Multi-Slice
SNP: Single Nucleotide Polymorphism
TMS: Transcranial Magnetic Stimulation
VA: Veterans Affairs
