# Supplementary material for: The effects of repetitive transcranial magnetic stimulation in older adults with mild cognitive impairment: a protocol for a randomized, controlled three-arm trial
Source: BMC Neurol. 2019 Dec 16;19:326. doi: 10.1186/s12883-019-1552-7 (PMC6912947; doi:10.1186/s12883-019-1552-7)
Supplement: Supplementary file 1 — Additional file 1. List of supplementary documents. The detailed study protocol (which includes details of data collection, AE monitoring, statistical considerations, and data management), exclusionary medications, and two rTMS manuals are available from the authors. [file 12883_2019_1552_MOESM1_ESM.pdf]

List of supplementary documents

The following documents and manuals are available from the authors

DSMB-approved Detailed Study Protocol

List of Exclusionary Medications

rTMS Operator’s Quick Guide User Manual

rTMS Operator’s Manual of Procedures

Corresponding author:

Joy L. Taylor, Ph.D.

VA Palo Alto Healthcare System (151Y)

3801 Miranda Avenue

Palo Alto, CA, USA 94304-1207

Tele: (650) 852-3457

Email: joyt@stanford.edu
