# Supplementary material for: The effects of repetitive transcranial magnetic stimulation in older adults with mild cognitive impairment: a protocol for a randomized, controlled three-arm trial
Source: BMC Neurol. 2019 Dec 16;19:326. doi: 10.1186/s12883-019-1552-7 (PMC6912947; doi:10.1186/s12883-019-1552-7)
Supplement: Supplementary file 2 — Additional file 2: Table S1. Schedule of enrollment, interventions, and assessments. [file 12883_2019_1552_MOESM2_ESM.pdf]

Table S1. Schedule of enrollment, interventions, and assessments

	Enrollment and Allocation		Intervention Phase		Follow-up Phase		
	In-person Screening	Baseline & Randomization	Sessions 1-10	Sessions 11-20	Immediate F/U 1 Week Post-Tx	3 Months Post-Tx	6 Months Post-Tx Close-out
Timepoint	t_1	t_2	$t_{3\text{-}7}$	$t_{8\text{-}12}$	t_{13}	t_{14}	t_{15}
**Enrollment**							
Informed Consent	X						
Mini-Mental State Exam, Logical Memory (Story A) I & II	X						
Clinical Dementia Rating Scale	X						
Cognitive Change Index, Geriatric Depression Scale, Alcohol Use	X						
Medical, Psychiatric & Seizure History	X						
Modified Hachinski Rating; PE/NE	X						
MCI classification and determination of Eligibility	X						
Allocation / Randomization		X					
**Interventions**							
Active DLPFC rTMS			↔	↔			
Active LPC rTMS			↔	↔			
Sham rTMS			↔	↔			
**Assessments**							
Demographics	X						
Motor Threshold (MT) determination	**Left MT**		**Right MT**				
California Verbal Learning Test (Primary outcome)		X			X	X	X
Secondary Cognitive and Mood assessments		X			X	X	X
Secondary Functional assessments (Study partner and participant)		X			X	X	X
MRI scan		X			X		
Blood Sample for Genomic DNA			X				
Plasma BDNF level			X	X			
Vital Signs	X		↔	↔		X	X
Concomitant Medications	X	X	↔	↔	X	X	X
TMS Safety Questionnaire	X		↔	↔			
rTMS Intervention Log			↔	↔			
TMS Blinding questionnaires (Participant and Operator)			X	X			
TMS Acceptability questionnaire				X			
Adverse Event monitoring		X	↔	↔	X	X	X
