# Supplementary material for: The effects of repetitive transcranial magnetic stimulation in older adults with mild cognitive impairment: a protocol for a randomized, controlled three-arm trial
Source: BMC Neurol. 2019 Dec 16;19:326. doi: 10.1186/s12883-019-1552-7 (PMC6912947; doi:10.1186/s12883-019-1552-7)
Supplement: Supplementary file 3 — Additional file 3. Informed consent documents. Research consent forms for the clinical trial and for an ancillary study involving urine specimens. [file 12883_2019_1552_MOESM3_ESM.pdf]

IRB Use Only
Approval Date: March 12, 2019
Expiration Date: March 12, 2020

Title of Study: Noninvasive Brain Stimulation for Mild Cognitive Impairment	ICF v1807 eProtocol 39859
Title of Consent (if different from Study Title):	
Principal Investigator: Joy L Taylor	VAMC: VA Palo Alto HCS

Noninvasive Brain Stimulation for Mild Cognitive Impairment

Informed Consent

Are you participating in any other research studies? _____ yes _____no

PURPOSE OF RESEARCH: Why is this study being done?

You are invited to participate in a research study to determine if repetitive Transcranial Magnetic Stimulation (rTMS)—a type of brain stimulation—can improve memory and thinking (cognition). Researchers at the VA Palo Alto and Stanford University expect to enroll 99 study participants, all of whom have signs of mild cognitive impairment (MCI). MCI means that a person's memory is impaired, but their overall thinking is not as impaired as it is with Alzheimer's disease dementia.

You may be a good fit for this study if you are between the ages of 55-90 and you experience memory problems that are consistent with MCI. You also need to have a spouse, friend, or relative—in other words, a "study partner"—who is willing to tell us about your functioning during the 7-month period of this study.

You have already completed a telephone interview about joining this research study. You are now receiving this consent form for you to read and think about before you participate further. The study staff will review the consent form with you and answer any questions you may have.

This study is being done together by researchers at VA Palo Alto and Stanford University.

VOLUNTARY PARTICIPATION

Your participation in this study is entirely voluntary. Your decision not to participate will not have any negative effect on you or your medical care. You can decide to participate now, but withdraw your consent later and stop being in the study without any loss of benefits or medical care you are entitled to.

DURATION OF STUDY INVOLVEMENT: How long will I be in the study?

If you are eligible and agree to participate, you will be in the study for about 7 months—including a 2- to 4-week period of receiving the study treatment, and a 6-month period of clinical follow-up.

PROCEDURES: What will happen if I take part in this research study?

Your participation would normally involve a total of 15 study visits (today's Initial Screening Visit, a Baseline Visit, 10 Treatment Visits, 1 Post-Treatment visit, and 2 Follow-up visits).

If you are eligible and agree to participate, you will be randomly assigned to receive ***either*** "real" rTMS ***or*** inactive/placebo rTMS. It is important to know that this study is a controlled clinical trial. Thus, not everyone in the study will receive real rTMS treatment. Those who receive placebo rTMS will serve as the control group. You have a 2/3 chance of getting the

active, real rTMS treatment. You have a 1/3 chance of getting the inactive, or placebo version of rTMS treatment.

Description of Study Activities

1. SCREENING VISIT (about 4 or 5 hours)

First, we will perform a few routine, clinical assessments to see if you are eligible and healthy enough to participate. The Screening Visit may be completed over one or two days:

- We will ask you for demographic information (such as your age, your occupation, your level of education).
- We will record your height, weight, and vital signs (blood pressure, heart rate, breathing rate and temperature).
- We will ask you about any medications that you are currently taking, or have taken in the past month, including vitamins or supplements. During the study, you will not be able to take any medications known to increase the risk of seizures. Your primary physician may adjust your medications as needed.
- A study clinician will ask you about your medical history. If the study doctor has concerns about your health, we may ask you to contact your primary doctor before enrolling in this study.
- You may have a brief physical and neurological examination.
- You will be given written tests of your memory and thinking, and you and your study partner will be asked questions about your daily functioning, mood, and your behavior. You can skip any questions you do not want to answer, and you can take breaks if needed.
- If you are a woman capable of becoming pregnant, a urine sample will be used to test for pregnancy. A positive test would exclude you from participation in this study. Additionally, it is important for you to tell us if you think you might be pregnant. The risks of rTMS on a pregnancy are unknown.
- We will take measurements using the rTMS machine to find your “motor threshold”. This is used to determine the settings that will be used for your study treatments.

In summary, the screening visit allows study staff to determine if you meet the eligibility requirements to be in the study and to make sure it is safe for you to undergo all of the procedures that are vital for the completion of the study.

2. BASELINE VISIT (4 to 5 hours)

<u>If you are eligible to be in the study, you can enroll and move onto the Baseline Visit.</u> The purpose of the Baseline Visit is to collect initial measurements of your memory and brain functioning. The Baseline Visit lasts 4 to 5 hours and may be completed over one or two days:

- You will be given written tests of your memory and thinking and a computerized attention test. You and your study partner will be asked to answer questions about your

daily functioning and your behavior. You can skip any questions you do not want to answer. You can take breaks if needed.

- You will undergo an MRI scan to take electronic pictures of your brain. MRI scanning is described in more detail later in this consent form.

3. TREATMENT PHASE (2 to 4 weeks)

The Treatment Phase involves the following procedures:

Randomization to Treatment:

- The study is a clinical trial, in which you will be randomly assigned to either the active "real rTMS" group or the inactive (placebo-control) group. Randomization is like flipping a coin, where one side of the coin is active and the other side is inactive. There is a 2/3 (66.7%) chance that you will receive the active "real rTMS" treatment, and a 1/3 (33.3%) chance you will receive the inactive/placebo treatment.
- If you are assigned to the active "real rTMS" treatment group, brief pulses of magnetic energy will be used to stimulate nerve cells in your brain. If you are assigned to the inactive (placebo-control) group, the exact same machine will be used; but the magnetic field reaching your brain will be reduced and your brain cells will not be stimulated.
- Neither you nor the study staff will know which treatment group you are in. The machine will always deliver the same treatment at each session. In case of an emergency, the study staff can find out which treatment you are receiving.

Treatment Scheduling:

- You will come in for 20 sessions of treatment, Monday through Friday. Each session may take up to 60 minutes. The treatment itself lasts about 20 minutes, plus another 10 to 30 minutes of preparation.
- You can do 2 or 3 sessions in the same day. If you want to do multiple sessions in the same day, you will have a 60- to 90-minute rest break between each session. If you prefer to do only 1 session a day, it is important that you complete 20 sessions within 30 calendar days.
- You may drive yourself to and from treatment visits and attend to your normal daily tasks before and after your treatment visits.

Procedures for Treatment Visits:

- During treatment, you will be reclined in a chair. You will be provided with ear plugs and headphones.
- You may close your eyes and rest while you sit still in the chair, but not sleep.
- At each visit, we will ask you about any changes in medicines you might be taking. We will also ask you about any changes in health or behavior since your last visit, which may or may not be related to the study treatment. We will ask you how much you have slept the night before and if you have taken any drugs or alcohol.

Description of each Treatment Session:

- You will be awake and alert while you will recline in the chair.

- You will wear a cap that is personalized for you. You will have electrodes placed on your forehead, and your head will be positioned in a holder so that it is correctly positioned.
- A metal coil in a plastic case will be held on the side of your head against your scalp. There will be a clicking noise as magnetic pulses are produced, but you will hear white noise through the headphones you will wear. We will give you earplugs to protect your hearing from the clicking sound.
- You may feel a tingling sensation on your head. Study staff will ensure you are as comfortable as possible during the study treatments.

At the first and last treatment visits:

- Blood samples of up to 4 teaspoons (20 mL) will be collected for research on biomarkers of response to brain stimulation (rTMS) and on the genetics of Alzheimer's disease (AD) and related conditions. Uses of samples in biomarker and genetic research is described in more detail later in this consent form.
- A saliva sample may be donated in lieu of blood for the genetic research, but blood samples are needed for the rTMS biomarker research.
- The total amount of blood drawn during study will be 40 mL (approximately 8 teaspoons).
- You will need to fast overnight (a minimum of 6 hours) prior to each blood draw.

4. POST-TREATMENT VISIT (4 to 5 hours)

1 to 3 days after your last treatment session, we want to repeat the tests you did at your baseline. The Post-Treatment Visit is very important so that we can measure changes in your memory, thinking, and brain function following treatment. At this visit you will repeat:

- Tests of memory, thinking, and attention, and questions about your mood and daily functioning.
- An MRI brain scan

5. FOLLOW-UP PHASE (about 2 to 3 hours per visit)

You will return two more times after the Post-Treatment Visit: 3 months after the Post-Treatment Visit, and 6 months after the Post-Treatment Visit:

- You will repeat the tests of your memory, thinking, and attention. We will again ask you about your mood and daily functioning.

MRI: Magnetic Resonance Imaging

An MRI machine uses a strong magnet and radiofrequency magnetic fields to take electronic pictures of your brain. The scanning procedure is very much like an X-ray or CT scan. You will be asked to lie on a long narrow couch for the scan (about 45 minutes) while the machine does its work. During this time you will not be exposed to x-rays, but rather a strong magnetic field and radiofrequency magnetic fields, which you will not feel. You will, however, hear repetitive tapping noises that arise from the Magnetic Resonance (MR) scanner. We will

provide earplugs or headphones that you will be required to wear. The space within the large magnet in which you lie is somewhat confined, although we have taken many steps to relieve the "claustrophobic" feeling.

IF YOU FEEL DISCOMFORT AT ANY TIME, NOTIFY THE OPERATOR AND YOU CAN DISCONTINUE THE EXAM AT ANY TIME.

The scans performed in this study are for specific research purposes and are not optimized to find medical abnormalities. The investigators for this project may not be trained to perform medical diagnosis. The investigators are not responsible for failure to find existing abnormalities with these MRI scans. However, on occasion the investigator may notice a finding on an MRI scan that seems abnormal. If this occurs, a doctor will be asked to look at the images to see if any medical follow-up is needed. If so, the investigator will contact you and recommend you inform your doctor about the findings. Because the images are taken using research settings they will not be made available for clinical purposes.

The MRI will take about 45 minutes of actual scanning, plus another ½ hour or so to prepare. The MRI will be performed at the Stanford University Medical Center at the Lucas Center, about 5 miles from the VA Palo Alto.

In the event that the scan has poor image quality and cannot be used, we will ask you if you would be willing to repeat the MRI scan.

Women of Child-bearing Potential

If you are a woman who is able to become pregnant, it is expected that you will use an effective method of birth control to prevent exposing a fetus to a potentially dangerous agent with unknown risk. If you are pregnant, you may not participate in this study. This is because the effects of rTMS on an unborn child (fetus) are not known. Similarly, you should not have an MRI scan if you could be pregnant. You understand that if you are pregnant, or if you become pregnant, you or your unborn child may be exposed to an unknown risk.

To confirm to the extent medically possible that you are not pregnant, you agree to have a pregnancy test done before the Baseline Visit. You must agree to avoid sexual intercourse or use a medically acceptable form of birth control birth while participating in the study. We will give you a list of acceptable forms. You must accept the risk that pregnancy could still result despite the responsible use of reliable method of birth control. You agree to notify the investigator as soon as possible of any failure of proper use of your birth control method, or if you become pregnant, either of which may result in your being withdrawn from the study.

Tissue Banking for Future Research

As part of this research, we would like to save any leftover samples of blood or saliva for future research. Your samples will be stored at the VA Palo Alto and will be used for future research on memory, cognition, and response to rTMS brain stimulation. Your samples will be stored until the sample is all used up. Both your samples and your research information will be labeled with a code that does not contain your name, initials, social security number, date of birth, or other ways that identify who you are. The research we conduct with your blood and/or

saliva is being done for research purposes only and we will not tell you or your doctor about the results of the research.

You may withdraw your permission for us to use your samples for future research at any time. Contact the Protocol Director, Dr. Joy L. Taylor, at (650) 493-5000 x 63102 to withdraw your permission. If you take back your permission, the research team can continue to use information about you collected before you decided to take back your permission, but they will not collect any information about you going forward and any remaining samples will be destroyed.

The research we conduct using your blood and/or saliva may result in inventions or discoveries that could be used to make new products or diagnostic or therapeutic agents. These inventions and discoveries may become financially valuable. You will not receive any money or other benefits from any commercial or other products that are made using your specimens.

Please initial below to indicate if you give permission for tissue banking.

_____ Yes, I give permission for my samples to be saved for future research, as set-forth above.

_____ No, I do not give permission for my samples to be saved for future research.

Tissue Sampling for Genetic Testing

Genetic tests will be conducted on your blood (or saliva) samples. The tests we plan to do will allow us to study (1) risk of progressive neurological diseases, such as Alzheimer's disease (AD), and (2) responsivity to brain stimulation (rTMS).

You will not be told the results of any of your genomic testing, even if some of the genes are related to AD risk. No information regarding your genetic or biomarker tests results will be entered in your medical records. If you are concerned about a potential genetic disorder, you should discuss this with your primary care doctor. You and your doctor may choose to test specifically for it, but this would require separate blood samples and would not be part of this research study.

Information from analyses of your coded samples and your coded medical information may be put into one of the National Institutes of Health (NIH) databases along with information from the other research participants and will be used for future research. If so, these databases will be accessible by the Internet. Only anonymous information from the analyses will be put in a completely public database, available to anyone on the Internet.

No traditionally-used identifying information about you, such as your name, address, telephone number, or social security number, would be put into the public database. While the public database will not contain information that is traditionally used to identify you, people may develop ways in the future that would allow someone to link your genetic or medical information in our databases back to you. For example, someone could compare information in our databases with information from you (or a blood relative) in another database and be able to identify you (or your blood relative). It also is possible that there could be violations to the

security of the computer systems used to store the codes linking your genetic and medical information to you.

However, your privacy is very important to us and we will use safety measures to protect it. Despite all of the safety measures that we will use, we cannot guarantee that your identity will never become known.

Sometimes patients have been required to furnish information from genetic testing for health insurance, life insurance, and/or a job. A Federal law, the Genetic Information Nondiscrimination Act of 2008 (GINA), generally makes it illegal for health insurance companies, group health plans, and employers with 15 or more employees to discriminate against you based on your genetic information.

We will protect the confidentiality of your samples and information about you. Your samples will be stored in a locked area, and all information about you will be stored in a locked file cabinet or on a password-protected secure computer.

PARTICIPANT'S RESPONSIBILITIES

As a study participant, your responsibilities include:

- Following the instructions of the investigators and study staff.
- Keeping your study appointments. If it is necessary to miss an appointment, please contact the investigators or research study staff to reschedule as soon as you know you will miss the appointment.
- Tell the investigators or research study staff about any side effects, doctor visits, or hospitalizations that you may have.
- Tell the investigators or research staff if you stop using birth control or think you might be pregnant.
- It is important that you not give false, incomplete, or misleading information about your medical history, including past and present drug use, because this could have serious consequences for your well-being. Stimulant drugs such as amphetamines and cocaine can increase the risk of a seizure. Alcohol and marijuana impair memory and cognition. If the study staff is concerned about your use of alcohol and other substances during your participation in this study, you may be asked to stop the study.
- Ask questions as you think of them.
- Tell the study doctor or research staff if you change your mind about staying in the study.

WITHDRAWAL FROM STUDY: Can I stop being in the study?

If you first agree to participate and then you change your mind, you are **free to withdraw** your consent and stop your participation at any time. If you decide to withdraw from the study, you will not lose any benefits to which you would otherwise be entitled to receive.

If you want to stop being in the study you should tell the investigators or study staff. You can

do this by phone by calling the Principal Investigator, Dr. Taylor at 650-493-5000 ext.63102, or by calling her study staff at 650-852-3457.

If you withdraw from the study, we ask that you return to the clinic site to undergo a final evaluation. This will involve the procedures normally performed at the Post-Treatment Visit.

Your participation may also be stopped by the study doctor or sponsor without your consent (for example, if the study team feels it is in your best interest, failure to follow the instructions of the study staff, or if the study is stopped). Should this happen, a member of the study team will contact you to discuss the reason for the discontinuation.

POSSIBLE RISKS, DISCOMFORTS, AND INCONVENIENCES

This study involves the following risks, discomforts, and possible inconveniences:

Risks of rTMS:

A few individuals receiving rTMS have had a seizure. This is based on a decade of research using rTMS. For example, in the treatment of major depression, the overall risk of seizure is estimated to be less than 1 in 1000 patient exposures (<0.1%) with the Neurostar coil. All seizures occurred during the rTMS session only. All the reported seizures resolved promptly on their own and none had any lasting effects or adverse impact. In summary, the risk of a seizure is low when rTMS is used the way it will be used in this study.

In the unlikely event that a seizure does occur, you will be closely monitored and treated for any medical or psychological consequences. Lab tests will be drawn and you will be seen by a neurologist as soon as possible. The facility where this and other rTMS studies are being conducted is fully equipped to safely handle a seizure. You will be given a letter regarding the seizure to share with your primary health care provider. The letter will indicate that the seizure during rTMS does not increase your risk for future seizures.

rTMS treatment can result in mild to moderate headaches in as many as 30 out of 100 (30%) patients. Some patients also report discomfort at the site of rTMS stimulation. This occurs in around 15 out of 100 of patients (15%). Headaches and site discomfort readily respond to acetaminophen or ibuprofen. Discomfort may improve over time or go away.

There is a low risk of dental pain during or immediately after an rTMS session. If this occurs, let your study doctor or nurse know. They may be able to move the rTMS coil slightly or give you a bite block to reduce or prevent the pain.

rTMS treatment may produce movement or tingling of the arm, leg, face, or scalp. You may also experience a temporary feeling of numbness in the face. There is a possible risk of hearing loss due to the light clicking sounds made by the device. You will wear ear protection during your treatment sessions. This should greatly reduce the possibility of hearing loss.

Rarely, people have fainted, particularly in the initial session of rTMS due to the newness of the procedure to them. If you feel faint or dizzy, be sure to let the study staff know. Our study staff are experienced, and you will be reclining in a chair to protect you from falling.

In some people, daily rTMS caused them to have increased energy, no need for sleep, and

rapid racing thoughts. This is called mania. If you notice these changes notify your primary physician and the study team.

The possibility of long-term risks is unknown. In previous studies, human and animal brains have shown no evidence of any kind of damage from rTMS. As with any investigational treatment, there may be unforeseen risks associated with the rTMS device. You will be informed of any new information that is developed during the study that might affect your willingness to continue your participation.

If you are taking any drugs that may increase the risk of having a seizure, you will need to be taken off those drugs before you can participate. You and your physician will need to discuss the feasibility of your discontinuing any such medication. Withdrawal from such drugs may cause discomfort or illness.

The rTMS operator will monitor you for ear protection, coil placement, and seizure activity during all rTMS sessions. Study staff will monitor you for side effects, and it is important that you report any side effect to study staff promptly. If you feel, or your study investigator feels, that the side effects are not well tolerated, treatment may be stopped altogether and you may be withdrawn from the study.

Risks of MRI:

Magnetic fields do not cause harmful effects at the levels used in the MRI machine. However, the MR scanner uses a very strong magnet that will attract some metals and affect some electronic devices. It is very important that you notify the researcher of any metal objects, devices or implants that are in or on your body before entering the magnet room. This includes biomedical devices such as pacemakers and aneurysm clips, prostheses, and any other metallic objects embedded in your body, such as bullets, buckshot, shrapnel, and any metal fragments from working around metal. All other metallic objects must also be removed from your person prior to entering the magnet room or approaching the magnet to prevent them from becoming a projectile or being pulled by the magnet. This includes keys, jewelry, body piercing, pocket knives, money clips, paper clips, safety pins, hair pins, and barrettes. In addition, objects such as watches, credit cards, and hearing aids could be damaged in the presence of the magnetic field. A locker will be provided for you to secure all your items and valuables.

Before you enter the scanner, we will ask you questions about whether you have any non-removable metal in your body. In addition, we may ask your permission to communicate with your physician to obtain information about your medical history if we need additional information to determine whether you should or should not have the MRI scan.

If you have any history of head or eye injury involving metal fragments, if you have ever worked in a metal shop, or if you could be pregnant, you should notify the operator / investigator. You should also notify the operator/investigator if you have any tattoos on your body. There is a small risk that areas with tattoo(s) could become warm, irritated and painful, and remain so for several days due to exposure to the RF electromagnetic field. If you are or

are trying to get pregnant, the effects of the scan on a fetus are unknown and, therefore, we will not perform the examination at this time.

There is a possibility that you will experience a localized twitching sensation due to the magnetic field changes during the scan. This is expected and should not be painful. Some of the radio frequency imaging coils, imaging software and devices being used in your scan are not approved by the FDA but are similar to counterparts that have been approved by the FDA. There is a small risk of heating from the cables associated with these devices. Please report any heating sensation immediately. Dizziness or nausea may occur if you move your head rapidly within the magnet.

IF YOU FEEL DISCOMFORT AT ANY TIME, NOTIFY THE OPERATOR AND YOU CAN DISCONTINUE THE EXAM AT ANY TIME.

Risks of Cognitive Testing and Questionnaires:

Memory and cognitive testing may cause some individuals to become upset, frustrated, or tired. You have the right to skip any questions that you feel uncomfortable in answering. You are free to take a break. Ask the study staff if you have any questions.

Risks of Blood Draws

There is a small risk of pain with a blood draw when the needle enters the skin. Bruising at the site of the needle stick may occur, but this is temporary. Some people may experience fainting or dizziness, and there is also a slight risk of infection at the site of the needle stick. To minimize these risks, experienced personnel will handle all the blood drawing procedures and sterile conditions will be maintained.

POTENTIAL BENEFITS: What are the benefits of taking part in this study?

We can't promise that you will get any benefits from taking part in this study. However, possible benefits may include improvement in memory, cognitive function, or mood. There are currently no effective treatments for MCI. The information obtained during this study is expected to help medical researchers learn more about how to treat Mild Cognitive Impairment (MCI). **WE CANNOT AND DO NOT GUARANTEE OR PROMISE THAT YOU WILL RECEIVE ANY DIRECT BENEFITS FROM THIS STUDY.**

ALTERNATIVES: What other choices do I have if I do not take part in this study?

The alternative to participating in this study is not to participate. Alternative choices include exercise and memory improvement classes. There are currently no effective treatments for MCI, aside from general health recommendations. Please ask your doctor about health recommendations for you.

PARTICIPANT'S RIGHTS

You should not feel obligated to agree to participate. Your questions should be answered clearly and to your satisfaction. You will be told of any important new information that is

learned during the course of this research study, which might affect your condition or your willingness to continue participation in this study.

ClinicalTrials.gov

A description of this clinical trial will be available on http://www.ClinicalTrials.gov, as required by U.S. Law. This Web site will not include information that can identify you. At most, the Web site will include a summary of the results. You can search this Web site at any time.

CONFIDENTIALITY

We will keep your name and all the information you tell us in this study as confidential as possible. We may publish the results of this study for others to read about, but you will not be identified in any articles about the study by name, social security number, address, telephone number, or any other direct personal identifier.

This research is covered by a Certificate of Confidentiality from the National Institutes of Health (NIH). The researchers with this Certificate may not disclose or use information, documents, or biospecimens that may identify you in any federal, state, or local civil, criminal, administrative, legislative, or other action, suit, or proceeding, or be used as evidence, for example, if there is a court subpoena, unless you have consented for this use. Information, documents, or biospecimens protected by this Certificate cannot be disclosed to anyone else who is not connected with the research except:

- if there is a federal, state, or local law that requires disclosure (such as to report child abuse or communicable diseases but not for federal, state, or local civil, criminal, administrative, legislative, or other proceedings, see below);
- if you have consented to the disclosure, including for your medical treatment; or
- if it is used for other scientific research, as allowed by federal regulations protecting research subjects.

The Certificate cannot be used to refuse a request for information from personnel of the United States federal or state government agency sponsoring the project that is needed for auditing or program evaluation by NIH National Institute on Aging, which is funding this project or for information that must be disclosed in order to meet the requirements of the federal Food and Drug Administration (FDA). Other federal agencies as required, such as the VA Office of Research Oversight and the VA Office of the Inspector General may have access to your information. Because this study involves an investigational device, the Food and Drug Administration may also have access to information about you collected in this study.

You should understand that a Certificate of Confidentiality does not prevent you from voluntarily releasing information about yourself or your involvement in this research. If you want your research information released to an insurer, medical care provider, or any other person not connected with the research, you must provide consent to allow the researchers to release it.

If, in an interview, you disclose that (a) you intend to harm yourself or someone else, (b) that a child had been abused or neglected, or (c) that an elder or dependent had been abused, we are required by California law to notify the appropriate authorities. The Certificate of Confidentiality will not be used to prevent us from notifying the appropriate authorities.

FINANCIAL CONSIDERATIONS

Payments. Will I be paid for taking part in this study? You will be compensated for your time and inconvenience. Generally, you will be paid $50 for a clinic visit, $50 for each MRI, and $25 for each of the 20 treatment sessions. There is no payment for the screening visit. If you complete the full study—which includes the Baseline, Treatment Phase, Post-Treatment visit, and the two Follow-up Visits—you will receive a $100 completion bonus. Thus, if you complete all of the study visits, you will receive a total of $900. If you withdraw from the study early, payment will be prorated based on the study visits completed. You will need to provide your social security number to receive payment.

You will be responsible for arranging transportation to and from all visits. If you live more than 40 miles from the VA Palo Alto, you can also receive payment for travel. The actual amount will depend on where you reside. For trips that you make to the VA—up to a maximum of 14 trips for study visits—we will reimburse you for each mile driven in excess of 40 miles one way, based on the government mileage rate.

Costs. You will not have to pay anything to be in this study.

Sponsor. This study is funded by a grant from the National Institute on Aging (NIA). The Department of Veterans Affairs is also providing financial support and material.

COMPENSATION for Research Related Injury

If you are injured as a direct result of being in this study, medical treatment will be available. If you are eligible for Veteran's benefits, the cost of such treatment will be covered by the VA. If not, the cost of such treatments may still be covered by the VA depending on a number of factors. In most circumstances, the treatment must be provided in a VA medical facility. No other form of compensation for injuries is available. However, by signing this form you have not released the VA from liability for negligence. For further information, you may call the Human Protections Administrator at (650) 493-5000, ext. 67593 or the V.A. Regional Counsel at (415) 750-2288.

CONTACT INFORMATION

Questions, Concerns, or Complaints. If you have any questions, concerns, or complaints about this research study, its procedures, risks and benefits, or alternative courses of treatment, you should ask the principal investigator, Dr. Joy Taylor at 650-493-5000 ext.63102. You should also contact her at any time if you feel you have been hurt by being a part of this study.

Independent Contact. If you are not satisfied with how this study is being conducted, or if you

have any concerns, complaints, or general questions about the research or your rights as a participant, and would like to speak with a person who is independent of the research, call the Stanford Institutional Review Board (IRB) at (650)-723-5244 or toll free at 1-866-680-2906. You can also write to the Stanford IRB, Stanford University, 3000 El Camino Real, Five Palo Alto Square, 4th Floor, Palo Alto, CA 94306.

Appointment Contact. If you need to change your appointment, please contact Nicole Strossman at 650-852-3457.

EXPERIMENTAL SUBJECT'S BILL OF RIGHTS

As a human subject, you have the following rights. These rights include but are not limited to the subject's right to:

- be informed of the nature and purpose of the experiment;
- be given an explanation of the procedures to be followed in the medical experiment, and any drug or device to be utilized;
- be given a description of any attendant discomforts and risks reasonably to be expected;
- be given an explanation of any benefits to the subject reasonably to be expected, if applicable;
- be given a disclosure of any appropriate alternatives, drugs or devices that might be advantageous to the subject, their relative risks and benefits;
- be informed of the avenues of medical treatment, if any available to the subject after the experiment if complications should arise;
- be given an opportunity to ask questions concerning the experiment or the procedures involved;
- be instructed that consent to participate in the medical experiment may be withdrawn at any time and the subject may discontinue participation without prejudice;
- be given a copy of the signed and dated consent form; and
- be given the opportunity to decide to consent or not to consent to a medical experiment without the intervention of any element of force, fraud, deceit, duress, coercion or undue influence on the subject's decision.

May we contact you (by phone or letter) about related studies that may be of interest to you?

_______ Yes, I would like to be contacted for future research opportunities.

_______ No, please do not contact me about future research opportunities.

Signing your name means you agree to be in this study and that you will receive a copy of this signed and dated consent form.

Signature of Participant Date

Print Name of Participant Date

Signature of Person Obtaining Consent

Signature of Person Obtaining Consent Date

Print Name of Person Obtaining Consent

HIPAA regulations require the participant to give separate written permission (signature) for the use of their protected health information.

Signature of Person Obtaining Consent HIPAA Authorization confirmation Date

☐ Confirm the participant signed the VA HIPAA Authorization (VA 10-0493)

Surrogate Consent

Note: *If the participant lacks the capacity to consent, consent must be obtained from a legally authorized representative (LAR) of the participant, with a description of the LARs authority to act for the participant.* ***The LAR must be (1)*** *a health care agent appointed by the participant in a dual power of attorney for health care or similar document;* ***(2)*** *a court-appointed guardian of the person,* ***or (3)*** *next-of-kin in the following order of priority: spouse, adult child (18 years or older), parent, adult sibling (18 years of age or older), grandparent, adult grandchild (18 years of age or older), or close friend.*

If the participant lacks the capacity to consent, obtain written consent from a LAR.

Signature of Legally Authorized Representative Date

Print Name of Legally Authorized Representative

Description of Representative's Authority to Act for Participant

Department of Veterans Affairs

RESEARCH CONSENT FORM

Title of Study: BDNF-mediated effects of rTMS on cognitive dysfunction in Veterans (45453)	
Title of Consent (if different from Study Title):	
Principal Investigator: **McNerney**	VAMC: VA Palo Alto HCS

BDNF-Mediated Effects of rTMS on Cognitive Dysfunction in Veterans

Informed Consent

What is this research about?

You are invited to participate in a research study to investigate signaling molecules in the blood and urine of participants receiving rTMS treatment for biomarkers of treatment response. You have already enrolled and consented to be part of an rTMS research study. The current study will utilize biological samples from your participation in the rTMS study to further assess for proteomic, genomic, and other signaling molecules as a function of rTMS treatment.

What is expected of me? (Procedures)

For this study, we will carry out biochemical analysis on blood samples already supplied by you as part of the rTMS study you are enrolled in. No extra blood will be required for participation in this study.

In addition, you will be asked to supply a urine sample before and after treatment, on the same day of the scheduled blood draw.

This study will completely overlap with your rTMS research study and thus is expected require only the donation of a urine samples twice. Blood sample will already be collected so no additional time is required.

Tissue Banking for Future Research

Research using tissues is an important way to try to understand human disease. You have been given this information because the investigators want to include your tissue in a research project and because they want to save the samples for future research. There are several things you should know before allowing your tissues to be studied.

Your tissue will be stored in a locked freezer in a locked laboratory at the VA Palo Alto for 6 years or until the samples have been used up. These samples will be stripped of identifying information and given a randomly generated code as an identification number (ID). The ID will be maintained by the Protocol Director and appropriately delegated staff, and will only be available to her in an encrypted, password-protected file on a physically sure, password protected computer at the VA Palo Alto.

You have the right to refuse to allow your tissues to be studied now or saved for future study. You may withdraw from this study at any time. Contact the Protocol Director, Dr. Windy

McNerney, at (650) 493-5000 x69129 to withdraw your permission. If you take back your permission, the research team can continue to use your information about you collected before you decide to take back your permission, but they will not collect any information about you going forward and any remaining samples will be destroyed.

The results of the study of your samples will be used for research purposes only and you will not be told of the test results.

Tissue Sampling for Genetic Testing

As part of the analysis on your samples, the investigators will do genetic testing. Genetic research is research that studies genes, including gene characteristics and gene versions that are transmitted by parents to children. Genetic research may include looking at information, such as personal appearance and biochemistry, gene sequences, genetic landmarks, individual and family medical histories, reactions to medications and responses to treatment. Genetic research raises questions about informing you of any results. Possible risks of knowing results include: anxiety; other psychological distress; and the possibility of insurance and job discrimination. A possible risk of not knowing includes being unaware of the need for treatment. The risks can change depending on the results of the research and whether there is a treatment or cure for a particular disease.

Sometimes patients have been required to furnish information from genetic testing for health insurance, life insurance and/or a job. A Federal law, the Genetic Information Nondiscrimination Act of 2008 (GINA), generally makes it illegal for health insurance companies, group health plans, and employers with 15 or more employees to discriminate against you based on your genetic information.

The results of the study of your sample from this project will be used for research purposes only, and you will not be told the results of the tests.

We will protect the confidentiality of your samples and information about you. Your samples will be stored in a locked area, and all information about you will be stored in a locked file cabinet or in a password-protected secure computer.

What are the possible risks or discomforts?

There is minimal risk for participation in this study. The only risk is for breach of confidentiality with your samples. However, this is a very small risk. Your samples will be labeled with a code that does not contain your name, initials, social security number, date of birth, or other ways that identify who you are. They will be stored in a locked freezer inside a locked laboratory. The research we conduct with your samples is being done solely for research purposes only and we will not tell you or your doctor about the results of the research. We will

use safety measures to protect your privacy, but we cannot guarantee that your identify will never be known.

Will I benefit from the study?

This research may result in inventions or discoveries that could be used to make new products or diagnostic or therapeutic agents. Otherwise you will not benefit from this study.

What are my alternatives to being in this study?

This is not a treatment study. There are no alternatives to participation in this study

Will I get paid?

You will not get paid for participating in this study

Will I have to pay anything?

You will not have to pay anything in this study

Do I have to be in this study?

Donation of urine and blood for this project is entirely voluntary. You may choose to provide one, none, or both for this study. Your decision will not impact your health care or have any other negative consequences.

Can I change my mind later and stop being in this study?

You may choose to stop providing samples for this study at any time without any loss of benefits in this study.

Will my information be protected from the public?

We may publish the results of this study for others to read about, but you will not be identified in any articles about the study as this study involves completely deidentified samples. Also, other federal agencies as required, such as the VA Office of Research Oversight and the VA Office of the Inspector General may have access to your information.

What happens if I think I've been hurt by being in this study?

If you are injured as a direct result of being in this study, medical treatment will be available. If you are eligible for veteran's benefits, the cost of such treatment will be

covered by the VA. If not, the cost of such treatments may still be covered by the VA depending on a number of factors. In most circumstances, the treatment must be provided in a VA medical facility. No other form of compensation for injuries is available. However, by signing this form you have not released the VA from liability for negligence. For further information, you may call the Human Protections Administrator at (650) 493-5000, ext. 67593 or the V.A. Regional Counsel at (415) 750-2288.

Who can I talk to if I have questions about the research, problems related to the study or if I think I've been hurt by being a part of the study?
If you have any questions, concerns or complaints about this research study, its procedures, risks and benefits, or alternative courses of treatment, you should ask the principal investigator, (Dr. McNerney, 650 493-5000 x69129). You should also contact her at any time if you feel you have been hurt by being a part of this study.

If you are not satisfied with how this study is being conducted, or if you have any concerns, complaints, or general questions about the research or your rights as a participant and would like to speak someone independent of the research team please contact the Stanford Institutional Review Board (IRB) at (650)-723-5244 or toll free at 1-866-680-2906. You can also write to the Stanford IRB, Stanford University, 3000 El Camino Real, Five Palo Alto Square, 4th Floor, Palo Alto, CA 94306.

What are my rights if I take part in this study?

You have the right to:
- be informed of the nature and purpose of the experiment;
- be given an explanation of the procedures to be followed in the medical experiment, and any drug or device to be utilized;
- be given a description of any attendant discomforts and risks reasonably to be expected;
- be given an explanation of any benefits to the subject reasonably to be expected, if applicable;
- be given a disclosure of any appropriate alternatives, drugs or devices that might be advantageous to the subject, their relative risks and benefits;
- be informed of the avenues of medical treatment, if any available to the subject after the experiment if complications should arise;
- be given an opportunity to ask questions concerning the experiment or the procedures involved;
- be instructed that consent to participate in the medical experiment may be withdrawn at any time and the subject may discontinue to provide samples without prejudice;

- be given a copy of the signed and dated consent form; and
- be given the opportunity to decide to consent or not to consent to a medical experiment without the intervention of any element of force, fraud, deceit, duress, coercion or undue influence on the subject's decision.

______________________________ ______________
Signature of Participant Date

____
Print Name of Participant

______________________________ ______________
Signature of Legally Authorized Representative Date

____
Print Name of LAR

____
Representative's Authority to Act for Subject

Person Obtaining Consent:

______________________________ ______________
Signature of Person Obtaining Consent Date

____
Print Name of Person Obtaining Consent

HIPAA regulations require the participant to give separate written permission (signature) for the use of their protected health information.

Person Obtaining Consent HIPAA Authorization confirmation:

☐ Confirm the participant signed the VA HIPAA Authorization (VA 10-0493)
