# Supplementary material for: The effects of repetitive transcranial magnetic stimulation in older adults with mild cognitive impairment: a protocol for a randomized, controlled three-arm trial
Source: BMC Neurol. 2019 Dec 16;19:326. doi: 10.1186/s12883-019-1552-7 (PMC6912947; doi:10.1186/s12883-019-1552-7)
Supplement: Supplementary file 4 — Additional file 4. Biological specimens manual. Appendix of procedures for the collection, processing, and storage of biological specimens for the current trial. [file 12883_2019_1552_MOESM4_ESM.pdf]

APPENDIX V.

SAMPLE COLLECTION, PROCESSING, STORAGE AND LABORATORY PROTOCOLS

A. Sample Collection Procedures

At the First Intervention Session (a.m. Visit 3), blood samples for genotyping, plasma-, and serum-derived biomarkers will be collected. Blood samples for plasma and serum biomarkers will be collected again at Intervention Session 19 (a.m. Visit 12).

FASTING OVERNIGHT (MINIMUM 6 HOURS) IS REQUIRED at both time points. Only water is permitted until blood draws are completed. The study visit schedule needs to include time for breakfast after the sample is drawn in the morning.

CONSENT: Before any samples are collected, the Study Coordinator will confirm the subject consented to biomarker collection per prior written informed consent. If the participant consented to biomarker collection, but is unable to provide a blood sample, a saliva sample can be collected in lieu of blood for the genetic research component.

TABLE 1 summarizes the sequence of tube collection at Intervention Sessions 1 and 19 (IS_1 and IS_19), and the instructions for the immediate handling of samples.

Table 1: Overview of Blood Sample Collection Procedures

IS 1 Order	IS 19 Order	Sample Uses	Tube Type	# Tubes x Volume (mL)	Total Amount (mL)	Instructions and
1	1	Whole blood: Genomic DNA banking<br>Plasma: Analysis of pre-post levels of BDNF at Intervention Sess 1 & 19<br>Buffy coat: Analysis of BDNF, APOE, COMT genotypes (Intervention Sess 1 only)	Purple top EDTA	1 x 10	10	Gently mix each tube by inversion, 10 -12 times;
2	2	Serum: Tissue Banking	Red top serum	1 x 10	10	Keep vertical and allow the blood to clot

* IS = intervention session

Blood samples need to be immediately delivered to Building 7 (Rooms A-106 and A-107) **for processing.**

Intervention Session 1 (Fasting, a.m. Visit 3) Sample Collection Procedures

Supplies Needed: 1 purple-top EDTA tube and 1 plain red-top tube

Steps

1. Labeling: Prior to drawing blood, write the “rTMS-MCI”, the Participant ID, date, and intervention session number on the side of each tube in permanent marker. Label the purple-top tube “1:Plasma.” Label the red-top tube “2:Sera”.

   NOTE: Use a ballpoint pen or permanent marker when completing the label.

2. Collect blood until each tube is full;
   - Tube #1:Plasma. One 10 mL EDTA (purple lavender top) tube of whole blood will be collected for plasma and for genetic biomarkers. Gently mix the tube by inversion, 10-12 times to assure that the EDTA anticoagulant is well-mixed with the blood.
   - Tube #2:Sera. One red top 10 mL tube of whole blood will be collected for serum; keep in a vertical position to allow blood to clot.
3. Estimate volume of blood in each tube and record on the **Biomarker Samples** form.
4. Immediately deliver the tubes to Building 7 so that the tubes of blood can be centrifuged within one (1) hour of collection. Note: There is time to escort the participant and study partner to the café for breakfast on the way from Outpatient Services to Building 7.
   - Deliver tubes to room A-107 for processing at the MIRECC research lab.

     Note: Samples of saliva, if collected in lieu of blood, need to be delivered to room 7A-106 for DNA extraction within 1 week of collection.
5. Fill out the **Genetic Lab Log** and the **Plasma & Serum Lab Log** as outlined on the next page.

Intervention Session 19 (Fasting, a.m. Visit 12) Sample Collection Procedure

Supplies Needed: 1 purple EDTA tube and 1 plain red-top tube

Steps

1. Labeling: Prior to drawing blood, write the "rTMS-MCI", the Participant ID, date, and intervention session number on the side of each tube in permanent marker. Label the purple-top tube "1:Plasma." Label the red-top tube "2:Sera."
   Note: *If the participant is discontinuing the intervention early,* but agrees to biomarker sample collection, write the date of the sample collection and the number of the most recently attended intervention session on the side of each tube.
2. Collect blood until each tube is full;
   - Tube #1:Plasma. One 10 mL EDTA (purple lavender top) tube of whole blood will be collected for plasma-derived biomarkers. Gently mix by inversion, 10-12 times.
   - Tube #2:Sera. One red top 10 mL tube of whole blood will be collected for serum banking; keep in a vertical position to allow blood to clot.

4. Deliver both tubes of whole blood to room A-107 immediately so that the tubes can be processed or centrifuged within one (1) hour of collection. Note: There is time to escort the participant and study partner to the café for breakfast on the way from Outpatient Services to Building 7.
5. Fill out the **Genetic Lab Log** and the **Plasma & Serum Lab Log** as outlined on the next page.

Plasma, Serum and Genetic Sample Logs

Study Coordinator (SC): Ensure that all fields on the Lab Log are complete. List in the comments section of the log any issues that occurred during the blood draw.

The **Plasma & Serum Lab Log** has the following columns:

- *Plasma drawn*: Yes or No (plasma = purple top)
- *Serum drawn:* Yes or No (serum = red top)
- *Date*: Date delivered to the lab
- *Project ID*: TAY0003
- *Participant ID*:
- *Session number*: normally, the Intervention Session number = 1 or 19;
- *Signature*: signature of the person who delivered the sample.
- *Comments:*

The **Genetic Lab Log** has the following columns:

- *Type of sample*: blood or saliva
- *Date*: date delivered to the lab
- *RDIS Project ID:* TAY0003
- *Participant ID*:
- *Signature*: signature of the person who delivered the sample
- *Consent* (Y/N): Did the participant give consent? (should be a "Y")
- *Comments:*

B. Processing and Storage Procedures for Samples

Overview. The amounts of BDNF in plasma will be quantified from the participant's two samples that are collected at Intervention Session 1and 19. Genomic DNA will be extracted from the participant's sample for genotype determination of three genetic variants: *APOE* e2/e3/e4, *BDNF* $Val^{66}Met$ (rs6265) and *COMT* $Val^{158}Met$ polymorphism (rs4680) and for genetic tissue banking. APOE, BDNF and COMT genotypes will be used in exploratory analyses of heterogeneity of response to rTMS. Sera will be banked to allow for additional analyses in the future.

Table 2 summarizes the purpose, processing, and storage of samples, and the relevant laboratory protocols.

Table 2: Biomarker Sample Processing and Storage Overview

Sample Purpose	Processing Summary	Storage*	Wet Lab Protocols
Measure pre- and post-rTMS levels of BDNF	Quantakine ELISA Kit	Plasma stored at -80C	See: "BDNF Protocols"
Determine BDNF, APOE, and COMT genotypes; Genomic DNA banking	DNA extraction: Qiagen DNeasy Kit	DNA stored at -80C	See: "Genotyping Protocols"
Sera: Tissue Banking		Sera stored at -80C	

*Freezer locations: Building 7, Rooms A-106 (DNA) and A-107 (plasma and serum), VA Palo Alto Health Care System (VAPAHCS), Palo Alto Division (PAD)

Processing of Plasma

Plasma Processing: 10 mL Lavender-Top Tube

1. If Visit 2, take out ~0.5ml blood for genomic DNA banking before spinning.
2. Centrifuge the tube at room temperature within one (1) hour of collection. Spin for 20 minutes using a Sorvall T 6000D Centrifuge (rotor H-1000B swinging bucket rotor) at 3000 rpm (1500 rcf) with the brake on, or in another centrifuge and rotor at a comparable rcf.
3. Using a STERILE pipette carefully transfer two 1mL aliquots of plasma into labeled 1.5mL plastic transfer tubes, and firmly cap with the white screw cap. Make sure the tubes are labeled with name of study, Participant ID, date of collection, intervention session number, and "plasma" on the transfer tubes using permanent marker.
4. Using a STERILE pipette carefully transfer the buffy coat layer (interface between plasma and blood) to a 1.5mL plastic transfer tube and firmly cap with the white screw cap. Make sure it is labeled with name of the study, Participant ID, date of collection, intervention session number and "BC" on the tube using permanent marker. This aliquot will be used for DNA extraction.
4. After the plasma has been transferred to the 1.5mL plastic tubes and capped, immediately transfer tubes to the -80C freezer in Building 7, A-107 (Dr. Salehi's lab). Place the remaining sample in the 13mL tube in the freezer -80C freezer for storage.
5. Fill out the **Plasma and Serum Lab Log** as outlined below.

Processing of Serum

Serum Processing: 10 mL Red-Top Tube

1. Allow the blood to clot for 30 minutes at room temperature in a vertical position.
2. Centrifuge the tube at room temperature within one (1) hour of collection. Spin for 20 minutes using the Sorvall T 6000D Centrifuge (rotor H-1000B swinging bucket rotor) at 3000 rpm (1500 rcf) with the brake on, or in another centrifuge and rotor at a comparable rcf.
3. Using a STERILE pipette carefully transfer two 1mL aliquots of serum into labeled 1.5mL plastic transfer tubes, and firmly cap with the white screw cap. Make sure the tubes are labeled with name of study, Participant ID, date of collection, intervention session number, and "serum" on the transfer tubes using permanent marker.
4. After the serum has been transferred to the 1.5mL plastic tubes and capped, immediately transfer tubes to the -80C freezer in Building 7, A-107 (Dr. Salehi's lab). Place the remaining of the sample in from the 13mL tube in the freezer -80C freezer for storage.
5. Fill out the **Plasma/Serum Lab Log** as outlined below.

Processing to Extract DNA

The DNA extraction procedure will begin within 3 hours of the blood draw. The Qiagen DNeasy Kit is used for DNA extraction. DNA will be stored in a -80C freezer in Building 7, A-106 (Dr. Hallmayer's lab). Fill out the **Genetics Lab Log** as outlined below.

Plasma/Serum and Genetic Lab Logs

Laboratory Staff: Ensure all fields on the worksheet are complete. List in the comments section of the worksheet any issues, e.g. a temperature excursion.

C. LABORATORY PROTOCOLS

BDNF Protocols

In this study, BDNF levels will be measured twice, prior to the first treatment session and again during the last treatment visit. An ELISA assay (Quantakine ELISA kits; R & D Systems) that has been optimized for plasma will be used, as described below. Sera will be banked to allow for additional analyses in the future.

BDNF ELISA Assay Protocol (adapted from R&D systems)

Reagents:

1. Wash Buffer – (remove crystals by warming to RT and mix gently) dilute 20mL of buffer into water into 480mL to make 500mL total.
2. Substrate Solution – Mix solutions A and B together in equal volumes (total volume = 200uL per well) within 15min from use. Protect from light.
3. BDNF Standard – Reconstitute with Calibrator Diluent RD6P to make a stock solution of 4,000pg/mL. Allow to sit for at least 15min with gentle agitation.
    - Make Serial Dilutions with **polypropylene tubes** – Add 300uL of the diluents in each tube, serially add 300uL to each tube from previous dilution for 1:2 ratio (usually 6-7 dilutions: 4000pg/mL; 2000pg/mL; 1000pg/mL; 500pg/mL; 250pg/mL; 125pg/mL; 62.5pg/mL; 0pg/mL)
4. Other solutions provided: Assay diluents, human BDNF conjugate, stop solution

Procedure (all solutions at RT and done in at least duplicate):

1. Prepare all reagents (see above)
2. Remove excess microplate strips from the plate frame, return them to the foil pouch and reseal
3. Add 100uL of assay diluents RD1S to each well
4. Add 50uL of the standard (step 3 above) or sample per well. Cover with adhesive strips and incubate for 2hr at RT
5. **Do not wash.** Add 100uL of human BDNF Conjugate to each well. Cover with the strip and incubate for 1hr at RT
6. Aspirate and wash each well 3x. Wash with 400uL wash buffer (step 1 above), make sure to completely remove liquid with each wash. After the last wash, invert the plate and blot it against clean towel.
7. Add 200uL substrate solution (step 2 above) to each well. Incubate 30min at RT, protect from light.
8. Add 50uL stop solution to each well. Gently tap the plate to ensure mixing. The color should change from blue to yellow.
9. Read with microplate reader at 450nm. If correction is available set to 540 or 570nm. If no correction is available, subtract readings at 540 or 570. This will correct for interference from the plate.

Data Processing:

1. Average duplicates or triplicates from each well and subtract the average zero standard optical density.
2. Create a standard curve using either the four parameter logistic curve-fit or plot the mean absorbance of the standards on the Y-axis against the concentration on the x-axis and draw a best-fit curve. Plot the log x log if this needs to be linearized (less accurate).
3. Compute concentration of samples using the standard curve.
4. Limit of detection is ~20pg/mL

Genotyping Protocols

Brain-Derived Neurotrophic Factor (BDNF): *G196A (val66met) polymorphism* (Ventriglia et al., 2002): Polymerase chain reaction (PCR) assays are performed in 8 µl volume reactions containing 10ng genomic DNA; 2.5 mM of each dNTP ; 0.2-0.6 µM of each primer; 0.8 µl of 10* buffer (Perkin-Elmer, Norwalk, CT); 1.5-2.5 mM MgCl2; and 0.2 units AmpliTaq Gold Polymerase (Perkin Elmer). PCR assays are performed in Thermocycler by denaturing for 10 minutes at 94° C, then 15 cycles of 30 sec at 94º C, 15 sec at 60º C, and 15 sec at 72º C. Another 20 cycles of 30 sec at 94º C, 15 sec at 65º C, and 15 sec at 72º C are performed, followed by a final extension step of 10 min at 72º C. Using the primers 5'-ACT CTG GAG AGC GTG AAT GG –3' and 5' –ACT ACT GAG CAT CAC CCT GGA-3' a 171bp product is amplified, followed by digestion with PmaCI restriction enzyme and agarose gel electrophoresis. The genotypes are assigned: GG – two bands 99 bp and 72 bp; GA – two bands 171 and 99 bp; AA – one band 171 bp.

COMT: The target 217bp COMT gene fragment is amplified using sense primer 5'-TCG TGG ACG CCG TGA TTC AGG-3' and the antisense primer 5'-AGG TCT GAC AAC GGG TCA GGC-3'. PCR reactions are carried out in a final volume of 15µl consisting of 50ng of genomic DNA, 50ng each of each primers, 7.5ul of Taq PCR Master mix (Qiagen, Cat.#201445) and10% DMSO. The PCR conditions include an initial denaturation step at 95C for 3 min, followed by 35 cycles of denaturation at 95C for 30s,annealing at 55C for 45 s and extension at 72C for 1 min, with a final extension of 10 min at 72C. The PCR products are digested at 37C for 3 hours with 5 U of the restriction enzyme NIa III (New England Biolabs, Cat#R0125S). Products are electrophoresed on a 10% Polyacrylamide gel(Acrylamide/bis-Acrylamide ratio 19:1) at 150 V for 40 min. A 10bp marker was used to measure the fragments size. The H allele, High activity Val-108, shows 2 bands, at 136bp and 81bp. The L allele, Low acitivity Met-108, shows 3 bands, at 96bp, 81bp and 40bp.

APOE: The target 244bp ApoE gene fragment was amplified using sense primer 5'-TAAGCTTGGCACGGCTGTCCAAGGA-3' and the antisense primer 5'-ACAGAATTCGCCCCGGCCTGGTACAC-3'. The PCR reaction are carried out in a final volume of 25µl consisting of 50ng of genomic DNA, 50ng each of sense and antisense primers, 12.5ul of Taq PCR Master mix (Qiagen, Cat.#201445) and10% DMSO. The PCR conditions include an initial denaturation step at 95C for 5 min, followed by 40 cycles of denaturation at 95C for 15s, annealing at 63C for 15 s and extension at 72C for 1 min30sec, with a final extension of 10 min at 72C. The PCR products are digested at 37C for 3 hours with 7.5 U of the restriction enzyme CfoI (Promega, Cat#R6241). The products were electrophoresed through 12% Polyacrylamide gel(Acrylamide/bis-Acrylamide ratio 19:1) at 180 V for 50 min. 10bp marker are used to measure the fragments size. E2 sample contains 91 and 83bp while E3 sample contains 91, 48 and 35bp fragments. E4 sample also shows 48 and 35bp fragment while contains unique 72bp fragment.
